# Molecular and functional characterization of the B-cell receptor in chronic lymphocytic leukemia-like monoclonal B-cell lymphocytosis

**DOI:** 10.1038/s41375-025-02797-y

**Published:** 2026-01-07

**Authors:** Andreas Agathangelidis, Anastasia Iatrou, Nikolaos Pechlivanis, Georgios Karakatsoulis, Laura Zaragoza-Infante, Chrysi Galigalidou, Palash Chandra Maity, Pamela Ranghetti, Julia Almeida, Blanca Fuentes-Herrero, Almudena Navarro, Miguel Alcoceba, Leily Rabbani, Birna Thorvaldsdottir, Lydia Scarfò, Alberto Orfao, Fotis Psomopoulos, Hassan Jumaa, Richard Rosenquist, Anastasia Chatzidimitriou, Paolo Ghia, Kostas Stamatopoulos

**Affiliations:** 1 Department of Biology, School of Science, National and Kapodistrian University of Athens, Athens, Greece; 2 Institute of Applied Biosciences, Centre for Research and Technology Hellas, Thessaloniki, Greece; 3 Institute of Experimental Cancer Research, University Hospital of Ulm, Ulm, Germany; 4 IRCSS Ospedale San Raffaele, Milan, Italy; 5 Translational and Clinical Research Program, Cancer Research Center (IBMCC, CSIC-University of Salamanca), 37007 Salamanca, Spain; 6 Cytometry Service, NUCLEUS, Department of Medicine, University of Salamanca (Universidad de Salamanca), 37007 Salamanca, Spain; 7 Institute of Biomedical Research of Salamanca (IBSAL), 37007 Salamanca, Spain; 8 Biomedical Research Networking Centre Consortium of Oncology (CIBERONC), Instituto de Salud Carlos III, 28029 Madrid, Spain; 9 Department of Hematology, Hospital Universitario de Salamanca-IBSAL, CIC- IBMCC (USAL-CSIC) CIBERONC, Salamanca, Spain; 10 Department of Molecular Medicine and Surgery, Karolinska Institutet, Stockholm, Sweden; 11 Università Vita-Salute San Raffaele, Milan, Italy; 12 Institut für Immunologie, Universitätsklinikum Ulm, Ulm, Germany; 13 Clinical Genetics and Genomics, Karolinska University Hospital, Stockholm, Sweden

**Keywords:** Chronic lymphocytic leukaemia, Cancer genomics

## To the Editor:

Chronic lymphocytic leukemia (CLL) is always preceded by monoclonal B cell lymphocytosis (MBL), characterized by the presence of circulating clonal B cells with a CLL phenotype, albeit at a lower number [1]. MBL, found in otherwise healthy individuals, is classified into two subtypes based on the number of circulating, “CLL-like” B cells [1]. CLL-type MBL (0.5–5 × 10^9^/l, previously termed “High-count MBL”) may progress to CLL requiring therapy at a rate of 1–4% per year [2], whereas low-count CLL-type MBL ( < 0.5×10^9^/l, previously termed “Low-count MBL”) is not generally associated with CLL, albeit may persist over time and even increase in size [2].

The clonotypic B-cell receptor (BcR) is critically implicated in the pathogenesis of CLL [3]. However, open questions abound regarding the BcR properties at the pre-malignant MBL stage, including reactivity, functionality and signaling capacity, that may be relevant for CLL ontogeny and evolution. Here, we comprehensively analyzed the BcR in low-count CLL-type MBL (*n* = 22) and CLL-type MBL (*n* = 14) (Supplementary Table 1), while also performing comparisons to CLL.

Clonality and BcR IG stereotypy analyses were performed on abundant BcR IG clonotypes, i.e., those with a frequency of >0.92%, each representing an independent CLL-like B-cell clonal expansion; relevant information is provided in Supplementary Results and Supplementary Tables 2 and 3. A monoclonal profile, i.e., clear dominance of a single BcR IG clonotype, was evident in 12/14 (85.7%) CLL-type MBL versus only 10/22 (45.5%) low-count CLL-type MBL cases (*p* < 0.05). Clonality profiles were further validated through analyzing available RNA-seq data with the MiXCR tool, leading to highly concordant results. Our findings are in line with previous studies showing that oligoclonality is more frequent in low-count CLL-type MBL and tends to decrease as it progresses to CLL-type MBL and CLL, which are predominantly monoclonal [4]. Recent studies showed that even CLL cases can exhibit multiple expanded CLL cell clones [5], indicating that leukemogenesis can occasionally be the result of competition between B cell clones with distinct BcR features.

Stereotypy analysis revealed a slightly stronger immunogenetic connection of CLL-type MBL to CLL, since 18/48 abundant clonotypes (37.5%) from CLL-type MBL were assigned to CLL stereotyped subsets compared to 62/175 (35.4%) from low count CLL-type MBL. Only 5/223 (2.2%) abundant BcR IGH clonotypes were assigned to major CLL stereotyped subsets [6]: 3 were typical of subset #2, including the dominant clonotype from HC-MBL_5 and 2 low-frequent clonotypes from LC-MBL_5 and LC-MBL_14 (frequencies: 2% and 2.3%, respectively). HC-MBL_ 5 progressed to CLL, albeit with no treatment requirement until the last follow-up in 2023. This case also carried an IGLV3-21 gene rearrangement typical of subset #2; however, of 101,069 sequences assigned to this clonotype, only 502 (0.5%) carried a SHM leading to the IGLV3-21^R110^ mutation that is associated with cell autonomous signaling in CLL [7]. LC-MBL_5 is still classified as low-count CLL-type MBL over a period of 13 years (2008–2021), yet exhibited an increase in clonal size (from 7.26 cells/μl in 2008 to 64.2 cells/μl in 2021). In contrast, LC-MBL_14 showed a decrease in clonal size over the same period (from 2.6 cells/μl in 2008 to 0.1 cells/μl in 2021, respectively). This is in line with our recent report that BcR IG clonotypes from poor-prognosis CLL subsets, including subset #2, can be identified up to 16 years before CLL diagnosis [8]. Hence, predicting MBL evolution based solely on immunogenetics remains challenging, with clone size probably playing a key role.

Intraclonal diversification analysis within the dominant BcR IG clonotypes was performed in the following groups: (i) oligoclonal MBL (mostly low-count CLL-type MBL clonotypes) (*n* = 19), (ii) monoclonal MBL (mostly CLL-type MBL clonotypes) (*n* = 12), (iii) CLL stereotyped subset #2 (*n* = 11), (iv) CLL stereotyped subset #4 (*n* = 18), and (v) non-stereotyped CLL (*n* = 34) (Supplementary Table 4). Overall, less pronounced intraclonal diversification was seen in oligoclonal MBL versus monoclonal MBL and all CLL groups, arguing for more temporally restricted and/or less intense antigen interactions (Supplementary Table 5 and Supplementary Fig. 1).

CLL displays a complex genomic landscape with recurrent, driver mutations in genes implicated in various biological mechanisms. Here, we confirm and extend our previous finding [3] that MBL shares few genetic CLL drivers, mostly at the subclonal level, with mutations appearing almost exclusively to CLL-like MBL. In particular, our analysis focused on 51 putative CLL driver genes (Supplementary Table 6) and detected a total of 11 variants (8/11 missense) (Supplementary Table 7), of which 10 (91%) were identified in 5 distinct CLL-type MBL samples. *TP53* was the most frequently targeted gene displaying 4 variants in 3 CLL-type MBL cases, followed by *EGR2* with 2 variants in 2 CLL-type MBL cases. Six of 11 (54.5%) variants exhibited a VAF > 20%; all 6 were detected in CLL-type MBL samples, yet none of the 2 cases with available clinical information progressed to CLL. Since driver mutations may have a smaller clinical impact in M-CLL compared to U-CLL, as exemplified by the limited impact of *TP53* mutations on TTFT in M-CLL [9], the lack of progression in MBL cases carrying driver gene mutations could perhaps be due to their mutated SHM status.

In the first ever transcriptomic comparison of MBL versus CLL, we report that while CLL-type MBL exhibited a signature of chronic inflammation that is typical also for CLL [10], low-count CLL-type MBL diverged significantly, with implications for a different ontogeny [11]. In specific, the FoxO, VEGF and Wnt signaling pathways, which are strongly implicated in CLL, were downregulated in low-count CLL-type MBL versus CLL (Supplementary Table 8 and Supplementary Fig. 2). Of note, the transcriptomic profile of low-count CLL-type MBL seems to resemble that of normal CD5^+^ B cells. The latter were found to exhibit an upregulation of genes implicated in the hematopoietic cell lineage as well as in antigen processing and presentation, while also displaying a downregulation of genes in the Notch, p53 and Wnt signaling pathways when compared to CLL cells [12].

**Fig. 1:**
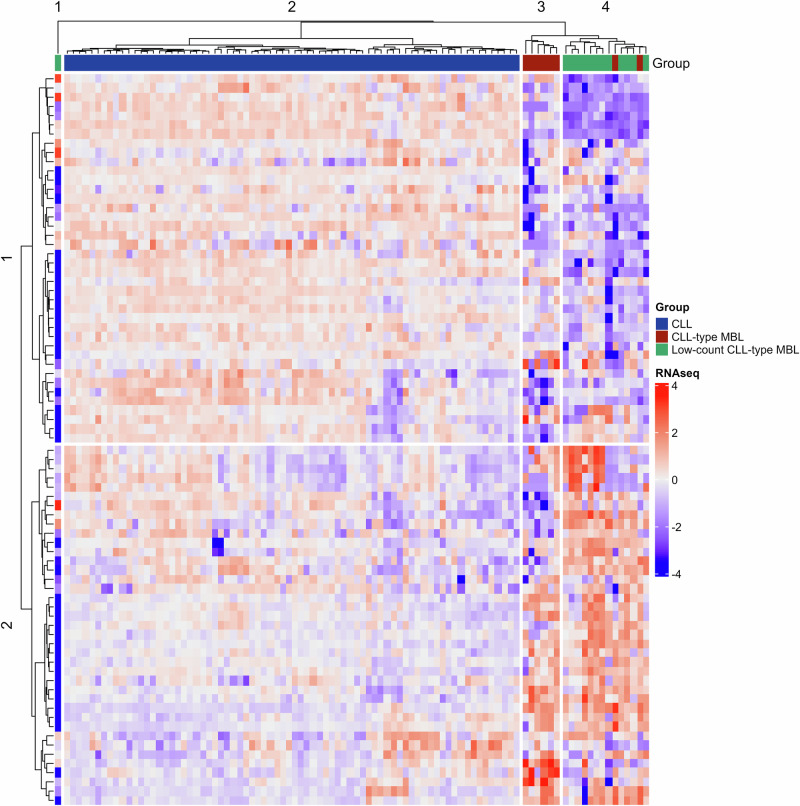
Heatmap of unsupervised hierarchical clustering based on RNA-seq data related to genes implicated in the BcR signaling pathway in low-count CLL-type MBL, CLL-type MBL and CLL. Columns represent samples and rows represent genes, respectively. Sample origin is depicted through a color code (green: low-count CLL-type MBL, red: CLL-type MBL and blue: CLL). The color bar indicates mRNA expression levels (dark red indicates high expression; dark blue indicates low expression). RNA-seq RNA-sequencing, BcR B cell receptor, CLL chronic lymphocytic leukemia, MBL monoclonal B cell lymphocytosis.

To assess the relevance of the BcR signaling pathway, we performed an unsupervised analysis using only relevant genes based on the KEGG 2021 database. This analysis provided further evidence for decreased BcR signaling capacity in low-count CLL-type MBL and CLL-type MBL versus CLL (Fig. 1); the relevance of this finding was further supported by its strong association with the size of the CLL clone. Relevant to mention, decreased BcR signaling capacity has also been reported in the normal B-cell compartment from individuals with IGHV-mutated MBL [13], alluding to a broad functional deregulation of B cells in MBL. A less pronounced role for BcR-associated processes in MBL versus CLL was also suggested by the enrichment of splicing events in the latter, mainly affecting the BcR and MAPK signaling pathways (Supplementary Table 9). Overall, these differences suggest differences in BcR signaling in MBL versus CLL that could influence cell behavior. This hypothesis is corroborated by our antigen reactivity studies, where recombinant monoclonal antibodies (rmAbs) from both MBL subtypes exhibited extensive auto/polyreactivity through mostly weak interactions, in clear contrast to the restricted and strong interactions of rmAbs from CLL subsets #1 and #4 (Supplementary Fig. 3). Of importance, the output of principal component analysis (PCA), depicted in Supplementary Fig. 4, led to highly concordant results.

**Fig. 2:**
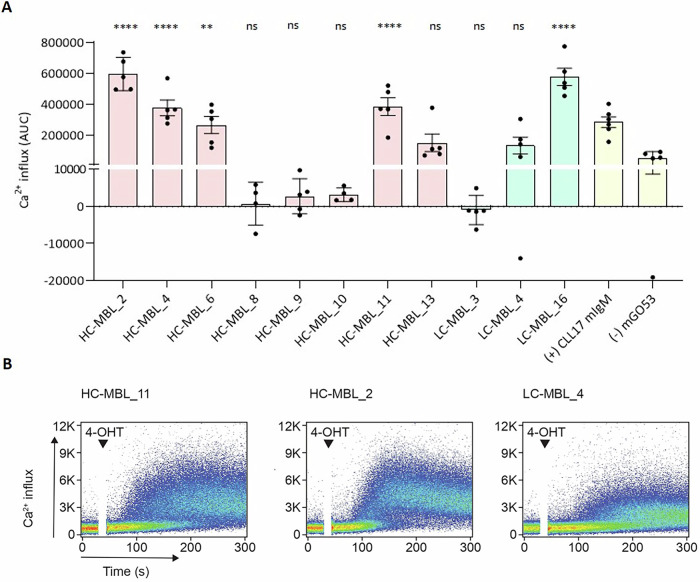
BcR from MBL possess antigen-independent cell-autonomous signaling capacity. **A** Mean difference of the area under the curve of Ca^2+^ mobilization of MBL-derived BcR. One-Way ANOVA (Bonferroni Correction) analysis was performed. *p ≤ 0.05, **p ≤ 0.01, ***p ≤ 0.001, ****p ≤ 0.0001. **B** FACS analysis for autonomous Ca^2+^ influx after the addition of 4-OHT in TKO cells expressing the ER^T2^–SLP65 fusion protein. Representative MBL BcR inducing strong (HC-MBL_11/HC-MBL_2) and weak (LC-MBL_4) cell-autonomous signaling. Addition of the stimulus (4-OHT) is indicated by black arrows. Identifiers for low-count CLL-type MBL and CLL-type MBL cases are abbreviated as LC-MBL and HC-MBL, respectively. BcR B cell receptor, MBL monoclonal B cell lymphocytosis, FACS Fluorescence-Activated Cell Sorting, 4-OHT 4-hydroxytamoxifen, TKO cells Triple knockout cells.

Besides interactions with “classic” antigens, autonomous BcR activation can constitute an oncogenic signal in primary CLL cells [14]. This mechanism was recently shown to be active in siblings of CLL patients exhibiting MBL [15], albeit it was less intense in MBL versus CLL, which could explain the lower levels of cell proliferation. Of note, this type of interaction was also identified in both subtypes of the present MBL cohort, where 7/11 (63.6%) analyzed rmAbs (CLL-type MBL, *n* = 5; low-count CLL-type MBL, *n* = 2) exhibited autonomous BcR signaling, while the remaining 4 (36.4%) were negative (CLL-type MBL, *n* = 3; low-count CLL-type MBL, *n* = 1) (Fig. 2). The intensity of autonomous BcR signals did not differ between MBL subtypes (mean AUC value for positive cases: 35,5671.44 in CLL-type MBL and 35,5804.3 in low-count CLL-type MBL, respectively). In terms of pathogens, *Mycoplasma pneumoniae* and Influenza A virus were recognized by 4 and 3 low-count CLL-type MBL rmAbs, respectively (Supplementary Fig. 5).

Statistical analysis for biological features that may correlate with the size of the CLL clone, i.e. the MBL subtype, revealed significant associations for age (*p* = 0.042), the clonality profile (*p* = 0.008), the genomic and transcriptomic profiles (*p* = 0.023 and *p* = 0.03, respectively) and, particularly, the BcR signaling profile (*p* = 0.002) (Supplementary Table 10). Multivariable analysis highlighted the clonality and BcR signaling profiles as independent risk factors for CLL clone size.

A limitation of the present work concerns the rather small size of the cohort, necessitating validation in larger series. Having said that, the consistency of our findings alludes to different processes that likely contribute to shaping a distinct BcR IG signaling profile in low-count CLL-type MBL versus CLL-type MBL and, particularly, CLL.

Taken together, on the evidence presented here, we argue that low-count CLL-type MBL may represent a state of clonal equilibrium, whereby no clone has such a proliferative/survival advantage as to dominate the repertoire. This can possibly be related to more adequate immune surveillance versus either CLL-type MBL or Rai 0-CLL, the low frequency of CLL genetic drivers and, the significant downregulation of the BcR signaling pathway.

## Supplementary information

Supplementary material

Supplementary tables

## Data Availability

All high-throughput data generated in this study are deposited at the European Nucleotide Archive (ENA) under the accession number PRJEB75741.
