# Supplementary material for: Molecular and functional characterization of the B-cell receptor in chronic lymphocytic leukemia-like monoclonal B-cell lymphocytosis
Source: Leukemia. 2026 Jan 7;40(2):454–8. doi: 10.1038/s41375-025-02797-y (PMC12875869; doi:10.1038/s41375-025-02797-y)

SUPPLEMENTARY MATERIAL

MATERIALS AND METHODS

Study group

Included in the study were 22 individuals with low-count CLL-type MBL and 14 individuals with CLL-type MBL, with a median follow up of 5 years (range: 6-15). The median ages for individuals with low-count CLL-type MBL and CLL-type MBL were 75 and 82 years, respectively. Additional information about the present MBL cohort is provided in **Supplementary Table 1**. None of the individuals had a family history of lymphoproliferative disorders. The study was approved by the Ethics Committees of the participating institutions and informed consent was obtained from all participants according to the Declaration of Helsinki.

Sample preparation, FACS analysis and cell sorting

Ten ml of peripheral blood were obtained from each participant and processed within 24 hours. The diagnosis of MBL was established by flow cytometry, as reported[1]. All samples were stained with anti-CD19, anti-CD5 and anti-CD20 antibodies and “CLL-type” MBL populations ($CD19^{+}CD5^{+}CD20^{dim}$) were flow-sorted, as reported[2]. The following antibody mixes were used: PE-cyanin7 (Cy7)–labeled anti-CD20, PE-cyanin5 (Cy5)–conjugated anti-CD5 and PE-Texas Red–conjugated anti-CD19 (Beckman Coulter). The flow-sorted CLL cells had a mean purity of 98.1% (**Supplementary Table 1)**.

Generation of NGS BcR IG sequence data and bioinformatics analysis

PCR amplification of BcR IG gene rearrangements, library preparation and high-throughput sequencing

Total RNA was extracted and 1 μg was used for cDNA synthesis with the SuperScript II RT kit (Life technologies, MA, USA). PCR amplification of the clonotypic IGHV-IGHD-IGHJ gene rearrangements was performed with a protocol developed by the Euroclonality-NGS Consortium (https://www.euroclonalityngs.org/usr/pub/pub.php), already applied in a recent study by our group[3]. PCR products were purified with the QIAGEN DNA purification kit (QIAGEN, Germany). Libraries were prepared using the NEBNext® Ultra™ II DNA Library Prep Kit (New England Biolabs, MA, USA). Paired-end sequencing was performed using the MiSeq Reagent Kit v3 (2x300bp) on a MiSeq Benchtop Sequencer (Illumina, CA, USA). Purity and size estimation of the libraries was performed on an Agilent Bioanalyzer using the Agilent High Sensitivity DNA kit (Agilent Technologies, CA, USA). The dsDNA BR and HS Assay kits were used for library quantification on a Qubit 3.0 fluorometer (ThermoFisher Scientific, MA, USA).

BcR IG gene repertoire analysis

BcR IG raw data and metadata analysis

An in-house, purpose-built bioinformatics algorithm was applied for data filtering. In detail, raw reads were filtered using the Illumina signal-processing software. Sequence assignment to samples was performed based on the incorporated Illumina indexes and adapter sequences were trimmed. Raw NGS data filtering was performed based on the following criteria: (i) length and sequencing quality of raw reads; (ii) quality of the variable heavy and light complementarity determining region 3 (CDR3); (iii) length and overall quality of the full-length sequences. BcR IG gene sequencing raw data were subjected to a strict filtering process and annotated with the use of IMGT/HighV-QUEST[4]. Metadata was processed with tripr[5].

BcR IG clonotype definition and assessment

All productive IG gene rearrangement sequences were analyzed and each unique nucleotide sequence was assigned to a clonotype, based on the recent criteria proposed by the EuroClonality NGS Working Group[6]. The most frequent clonotype of each sample, i.e. the one comprising the highest number of identical IG gene rearrangement sequences, was defined as dominant[6].

For the purposes of clonality assessment, we evaluated abundant clonotypes, i.e. those displaying a frequency of >0.92%. This cut-off was set on the basis of statistical analysis performed in the context of a recent publication,[3] where we assessed the frequency distribution of clonotypes in individuals with MBL versus healthy individuals. According to our results, BcR IG clonotypes in healthy individuals showed a minimal, if any, probability to display a relative frequency higher than 0.92%. Hence, all clonotypes that present with a frequency of >0.92% can be considered as relevant to MBL. A monoclonal profile was characterized by the presence of a single abundant clonotype dominating the repertoire, while cases with oligoclonal profiles exhibited a small series of dominant clonotypes with comparable frequencies.

Even though B cells were sorted based on the expression of a “CLL-like” phenotype and, moreover, a frequency cut-off was applied to remove all irrelevant, polyclonal B cell expansions, the possibility that some abundant clonotypes may represent expansions of normal B cells cannot be *a priori* excluded.

Analysis of intraclonal diversification

Analysis of intraclonal diversification within the IG genes was performed using IgIDivA[7]. In brief, this analysis entailed: (i) identification of somatic hypermutation (SHM) pathways connecting the dominant clonotype and closely related clonotypes; (ii) quantification of metrics related to intraclonal diversification; and (iii) statistical comparisons between groups. The average number of evaluated clonotypes was 158 for CLL-type MBL and 184 for low-count CLL-type MBL, respectively. These highly

comparable numbers argue that any differences in intraclonal diversification complexity between the MBL subtypes could not be attributed to cell numbers and/or clonality status.

Intraclonal diversification in each analyzed sample was performed using the dominant clonotype (main nucleotide variant) as a reference and comparing all immunogenetically related clonotypes (subclonotypes), i.e. those with a similar somatic hypermutation (SHM) pattern, yet with fewer or additional SHMs against the dominant clonotype[7]. After establishing the connections between all individual clonotypes, 4 mutational pathways were selected as representative of the intraclonal diversification process: (i) the "negative" pathway (NP), defined as the pathway with subclonotypes that have common, yet less SHMs compared to the dominant clonotype; (ii) the "most relevant" pathway (MRP) that concerns connected clonotypes with the highest cumulative number of BcR IGH or BcR IGK/L sequences; (iii) the "longest" pathway (LP), which contains the largest number of connected clonotypes; (iv) the "longest mutational" pathway (LMP) that contains connected clonotypes with the highest total number of somatic hypermutations (SHMs).

These pathways were used for the calculation of metrics specific for the analysis of intraclonal diversification, such as relative reads convergence (RRC), end nodes density (END), maximal pathway length (MPL), maximal mutational length (MML) as well as established Graph Theory metrics, such as average degree (ADe) and average distance (ADi). More specifically, the RRC metric focuses on the MRP pathway and represents the tendency for the BcR IG clonotypes to either accumulate to the dominant clonotype or to form clonotypes with distinct SHM patterns. The END metric reflects the randomness or specificity of the SHM acquisition. The MPL corresponds to the number of connected clonotypes being included in the identified LPs, providing information regarding the overtime evolution of the SHM process. The MML shows the maximum level of additional SHMs in the LMPs. Finally, the ADe provides the mean number of connections of each clonotype, whereas ADi represents the mean number of clonotypes being located in between any individual pair of clonotypes through assessing all possible mutational pathways

connecting those clonotypes. In brief, a high level of intraclonal diversification is reflected in a low value for the END metric, as well as high values for all other metrics.

To avoid considering PCR amplification and/or NGS artefacts rather than clonotypes with actual mutations, we applied the following criteria: (i) we removed clonotypes with less than 10 sequences as well as clonotypes exhibiting very low levels of ongoing SHM.

BcR IG stereotypy investigation

BcR IG stereotypy analysis was undertaken as reported[8] for all abundant BcR IGH clonotypes from this study as well as 39,386 Sanger-derived IGHV-IGHD-IGHJ gene rearrangement sequences from patients with CLL deposited in the IMGT/CLL-DB.

Targeted re-sequencing and bioinformatics analysis

DNA extraction and library preparation

The QIAamp DNA Micro kit and the QIAamp DNA Blood Mini kit (Qiagen, Germany) were used for genomic DNA extraction in low-count CLL-type MBL and CLL-type MBL samples, respectively. DNA quantity and quality were assayed using the Qubit dsDNA HS Assay Kit (Life Technologies, MA, USA). Library preparation was performed using the KAPA HyperPlus kit (Roche, Switzerland). xGen Duplex Seq adapters were included as unique molecular identifiers. Multiplexed libraries were hybridized to capture probes of the GMS Lymphoid gene panel (Twist Bioscience, CA, USA)[9]. Paired-end 150bp sequencing was performed on a NovaSeq 6000 (Illumina, CA, USA).

Bioinformatics analysis

Demultiplexing was conducted with bcl2fastq version 2.20. Fastq files were processed using the Balsamic pipeline version 7.2.5[10] and reads were mapped to the hg19 human reference genome. Aligned reads

were used to detect SNVs, indels and MNPs through the following procedure. At first, UMI barcodes were added as a separate tag (RX tag) to the alignment files. Tagged bam files were sorted and checked for the correct assignment of all mate-related flag fields using 'sortBam' and 'SetMateInformation' of fgbio version 2.1.0 (http://fulcrumgenomics.github.io/fgbio/), respectively. Subsequently, reads were grouped based on their UMI barcodes using the 'GroupReadsByUmi' tool of fgbio in paired mode. This step was followed by sorting the grouped reads and generating consensus reads using 'sortBAM' and 'CallDuplexConsensusReads' tools of fgbio with mean reads 1,0,0. Then, consensus reads were filtered with the fgbio 'FilterConsensusReads' tool and fastq files were generated using the 'samtools fastq' version 1.17[11]. The bwa-mem2 version 2.2.1 was applied to align the consensus reads to hg19. These alignments were used as input for the VarDictJava version 1.8.3[12], a sensitive variant caller, with minimum allele frequency of 0.01. All scripts can be found on GitHub (https://github.com/clinicalgenetics/BcR_in_MBL).

Annotation was limited to variants located within the 51 genes overlapping between the 252 genes included in the GMS Lymphoid gene panel[9] and the 103 genes characterized as genetic drivers in 3 recent genomic studies in CLL[13–15] (**Supplementary Table 6**). Gene variants were annotated with the ensembl-vep version 111.0[16] and filtered-in when meeting the following criteria: (i) single nucleotide variant (SNV), insertion-deletion (indel) or multiple nucleotide polymorphism (MNP); (ii) missense, indels or splice variants; (iii) maximum gnomAD population frequency of < 0.01; (iv) total sequence depth of ≥ 50; (v) alternate allele depth of ≥ 3; (vi) VAF ≥ 0.02; (vi) classification as pathogenic or likely pathogenic in ClinVar; and (vii) deemed functionally relevant by majority of SIFT, PolyPhen-2, AlphaMissense and CADD.

Transcriptome analysis

Transcriptome analysis was performed on polyA-selected RNA from 16 low-count CLL-type MBL and 8 CLL-type MBL cases as well as public RNA-seq data from 74 CLL cases[17] (ICGC database, Dataset ID:

EGAS00001000374). Total RNA was extracted from flow-sorted “CLL-type” MBL cells ($CD19^{+}CD5^{+}CD20^{dim}$) using either the QIAamp RNA Blood Mini Kit (Qiagen, Germany) for CLL-type MBL cases or the AllPrep® DNA/RNA Micro Kit (Qiagen, Germany) for low-count CLL-type MBL cases.

Library preparation

In CLL-type MBL samples, 1 µg of total RNA was used for library preparation, while 100 ng of total RNA were processed in low-count CLL-type MBL cases. To minimize the potential impact of the different RNA input, we used quantity-based kits for library preparation, to ensure the generation of comparable gene expression profiles. In detail, the NEBNext ® Ultra™ II Directional and the NEBNext® Single Cell/Low Input RNA Library Prep Kits (New England Biolabs, MA, USA) were used for CLL-type MBL and low-count CLL-type MBL samples, respectively. We also checked whether differences in the RNA input could affect the complexity of the results, i.e. the number of genes with quantifiable expression levels, and observed no differences between the two MBL subtypes. Moreover, no gene expression differences were evident in basic cellular mechanisms, arguing against experimental biases. Library quantification was performed with the KAPA SYBR FAST Universal qPCR kit (KAPA Biosystems, MA, USA) and libraries were paired-end sequenced on a NextSeq 500 Illumina platform (Illumina, CA, USA).

Bioinformatics analysis

Raw reads were quality-controlled using the FastQC software (https://www.bioinformatics.babraham.ac.uk/projects/fastqc/) (Babraham Institute, Cambridge, UK). Subsequently, reads were trimmed and filtered using the Trim Galore! suite (wrapper for cutadapt and FastQC) (https://www.bioinformatics.babraham.ac.uk/projects/trim_galore/) (Babraham Institute, Cambridge, UK) and aligned against the UCSC hg19 reference genome using HISAT2[18]. Differential gene expression analysis (mean fold change 1, adjusted p value <0.05) was performed with the Limma

package[19]. Gene enrichment analysis was performed with Enrichr[20] ($p<0.05$), using the KEGG 2021 gene database.

Alternative gene splicing analysis

Alternative splicing patterns were analyzed using SGSeq[21]. Eleven types of alternative splicing events were considered, including alternative 5' splice site (A5SS), alternative 3' splice site (A3SS), skipped exon (SE), 2 consecutive exon skipped (2SE), retained intron (RI), mutually exclusive exons (MXE), alternative first exon (AFE), alternative last exon (ALE), alternative start other than AFE (AS), alternative end other than ALE (AE), and unknown/mixed type. Differential gene splicing at the individual event level was performed with the Limma package[19]. Events exhibiting a mean fold change of 2 (>2 or <-2) and an adjusted p-value of <0.05 were considered as significantly differentially distributed among groups. Finally, enrichment analysis for genes exhibiting significant differences in their splicing patterns among the different groups was performed with Enrichr[20] (p-value <0.05) using the KEGG 2021 gene database.

Generation of recombinant monoclonal antibodies and reactivity profiling

Production of recombinant monoclonal antibodies

Recombinant monoclonal antibodies (rmAbs) were prepared for 16 MBL cases (10 CLL-type MBL and 6 low-count CLL-type MBL, respectively) and 13 CLL cases belonging to stereotyped subsets #1 (n=3), #2/#169 (n=4) and #4 (n=3), or non-stereotyped U-CLL (n=2) and M-CLL (n=1).

To produce each rmAb, we co-transfected 10 µg of each vector containing the dominant BcR IG heavy and light chain gene sequences into HEK293T cells using linear polyethylenimine (Sigma-Aldrich, MO, USA). Seven days after transfection, cell culture supernatants were collected and rmAbs were harvested using filter concentrators with a 100 kDa molecular weight cut-off (Millipore, MA, USA). The production of

soluble pentameric IgM was confirmed by quantitative IgM ELISA and Western blotting, as previously described[22].

Protein microarray and data analysis

Autoantigen reactivity of the produced rmAbs was tested with the OmicsArray™ protein microarray chip (GeneCopoeia Inc., MD, USA) using the rmAbs as primary antibodies. All autoantigens and four control proteins of the array were printed in duplicates onto nitrocellulose film slides. The primary antibodies were incubated with the autoantigen arrays. Stringent washing conditions were used to ensure optimum binding specificity. Bound rmAbs were measured with cy5-conjugated anti-human IgM (1:2000, Jackson ImmunoResearch Laboratories, PA, USA), using a GenePix® 4000B scanner. The fluorescent signals from the array were analyzed using the GenePix Pro v7.0 software to obtain raw data, including foreground signals, background signals, SNR (signal-to-noise ratio) and flags (poor-quality data identified by the software). Next, the net fluorescence intensity (NFI) value was calculated (NFI = foreground median - background median).

Microarray data analysis was performed in R v4.2.0. To minimize biases due to technical limitations, raw data was normalized against the controls based on the CPM (counts per million) methodology. Following that, unsupervised hierarchical clustering was performed to investigate the existence of distinct BcR IG reactivity signatures. To this end, the Euclidean distance was used for the generation of distance metrics and the ward.D2 method[23] for the unsupervised clustering of samples and autoantigens, respectively. In addition, principal component analysis (PCA) was applied on the normalized data to further investigate data/sample clustering. Finally, identification of statistically significant differences in terms of BcR IG reactivity patterns between samples from MBL subtypes and CLL was conducted using the Limma package[19]. Differences exhibiting p-values of < 0.05 were considered as statistically significant.

ELISA experiments against autoantigens and microbial antigens

ELISA experiments were performed with either in-house indirect ELISA or commercial ELISA kits. In more specific, reactivity against DNA, human skeletal muscle actin (globular: G-actin), myosin, human thyroglobulin (Tg), β-amyloid peptide (1-40), carbonic anhydrase, the F(ab')2 fragment of human serum IgG, the non-self hapten trinitrophenyl (TNP)-BSA and the oxidative stress marker malondialdehyde (MDA)-BSA was tested through in-house indirect ELISA.

G-actin, myosin and TNP–BSA were prepared as described previously[24]. Human thyroglobulin (Tg) was extracted from the healthy portion of thyroid glands dissected from patients operated for papillary thyroid cancer (kindly provided, under informed consent, by Dr Kakaviatos, Athens General Clinic), as previously described[25]. Human IgG F(ab')2 fragments derived from an IVIg preparation (Kiovig) were prepared, as already reported[26]. Carbonic anhydrase from bovine erythrocytes, β-amyloid human peptide (1-40) sodium salt and deoxyribonucleic acid sodium salt from calf thymus (native DNA type I) were purchased from Sigma-Aldrich (Munich, Germany). MDA-BSA was purchased from Cell Biolabs Inc. (San Diego, CA, USA).

Polystyrene microtitre plates were coated for 1 hour at 37°C followed by overnight incubation at 4°C with G-actin (10 μg/ml), Tg (10 μg/ml), myosin (10 μg/ml), carbonic anhydrase (10 μg/ml), TNP–BSA (10 μg/ml), β-amyloid peptide (2 μg/ml), F(ab′)2 fragments (2 μg/ml) in carbonate-bicarbonate buffer (0.1M pH 9.6) or DNA (10 μg/ml) and MDA-BSA (2 μg/ml) in PBS (pH 6.85). Next, plates were washed thoroughly with PBS and then saturated for 1 hour at 37°C with PBS containing 1% BSA (Sigma-Aldrich, MO, USA). RmAbs were used as primary antibodies in duplicates diluted in PBS containing 0.1% Tween and 1% BSA (PBS-T-BSA) at a concentration of 15 μg/ml and were allowed to react with each antigen of the panel for 2 hours at 37°C. Next, alkaline phosphatase conjugated anti-human IgM (Sigma-Aldrich, MO, USA) was used as secondary antibody at a concentration of 600 ng/ml in PBS-T-BSA for 2 hours at 37°C followed by washes

with PBS containing 0.1% Tween (PBS-T) and PBS alone. P-nitrophenylphosphate (pNPP) substrate was added for 30 minutes followed by the measurement of the OD values at 405 nm.

Reactivity against Cytomegalovirus, Epstein-Barr virus early antigen, Influenza Virus A, *Mycoplasma pneumoniae*, *Helicobacter pylori* and *Candida albicans* was assessed using commercial ELISA kits (IBL International, Germany). Microplates were pre-coated with the specific antigens and the rmAbs were added in duplicates at a concentration of 30 μg/ml followed by incubation for 1 hour at 37°C. After washing the wells to remove all unbound sample material a horseradish peroxidase (HRP) labelled conjugate was added for 30 minutes at room temperature. During a second washing step unbound conjugate was removed. The immune complex formed by the bound conjugate was visualized by adding tetramethylbenzidine (TMB) substrate followed by the addition of sulphuric acid to stop the reaction. Absorbance at 450/620 nm was read using a TECAN-Spark microplate reader (Salzburg, Austria).

Analysis of autonomous BcR signaling capacity

Three low-count CLL-type MBL and 8 CLL-type MBL cases were analyzed for autonomous BcR signaling capacity, as reported[27,28]. In brief, the dominant IG heavy and light chain clonotypes from each case were amplified and cloned by ARTISAN PCR, and then transduced into the BcR-deficient, pro-B cell line derived from the *RAG2/λ5/SLP65* triple knockout (TKO) mouse model. Autonomous signaling capacity was assessed as described before[27,28]. Briefly, the dominant heavy and light chain BcR IG sequences were cloned into a retroviral vector as a single ORF joined by cleavable P2A peptide, which was then expressed in ER^{T2}-SLP65 expressing TKO cells; BcR IG expression was validated through FACS. Autonomous signaling was analyzed through the estimation of Ca^{2+} influx upon stabilizing the ER^{T2}-SLP65 through the addition of 2 μM 4-Hydroxytamoxifen (4-OHT). To confirm the competence of autonomous BcR signaling, cells were additionally treated with anti-IG IG light chain (Igκ/λ) antibodies resulting in BcR cross-linking. Data was plotted in FlowLogic 8.4 and analyzed for area under the curve (AUC) for median Ca2+ influx response.

For the purposes of the analysis, data were also statistically compared against a non-autonomous BcR (mGO53) and an autonomously active, CLL subset #2 BcR, which served as negative and positive controls, respectively. Finally, MBL cases were classified as: (i) positive (recurring autonomous BcR signaling in 3-5 independent transductions), or (ii) negative (autonomous BcR signaling in ≤2 independent transductions).

Statistical analysis

Descriptive statistics were utilized for analyzing the NGS data. In the case of qualitative variables, the analysis included counts and frequency distributions. For quantitative variables, statistical measures included the mean and median values. To examine potential associations between two categorical variables, the chi-square and Fisher's exact tests were conducted. The Kruskal-Wallis test was applied to examine whether the distribution of a continuous variable differed between the levels of a categorical variable. Finally, to test associations between two continuous variables, the Pearson's correlation coefficient as well as the linear regression were assessed. For all comparisons, a significance level of p=0.05 was set. To maintain the significance level in multiple testing, the correction of Benjamini-Hochberg at each post-hoc comparison was used. All analyses were conducted in R (v. 4.3.1).

In more specific, the analysis of BcR reactivity data was based on the use of either the paired t-test or the non-parametric Mann-Whitney U test. Analyses were performed with GraphPad Prism 9 software (San Diego, CA). Significance was set at p=0.05. All statistical analyses were performed with SPSS V-22 and R V-3.4.3.

Statistical analysis of Ca^{2+} influx in the context of autonomous BcR signaling was performed through the calculation of the mean difference of the area under the curve (AUC) between each MBL case and the negative control (healthy PBMCs) derived from several individual replicates.

Finally, in the case of the data association analysis, descriptive statistics, frequencies and relative frequencies were used for the categorical variables, whereas median values were used for the numeric

ones. For the inferential analysis, the primary outcomes were the MBL subtype and the size of the CLL clone. We first examined potential correlations between the risk factors. To this end, we used the Kendall's correlation coefficient for comparisons between two numeric variables, the chi-squared or Fisher's exact test for comparisons between two categorical variables and the Kruskal-Wallis test for comparisons between a numeric and a categorical variable. Furthermore, we applied logistic regression to investigate the risk factors associated with the MBL subtype, whilst linear regression for the risk factors associated with the size of the CLL cell clone. We conducted both univariable and multivariable analyses. For the multivariable analysis models, we first selected the significant risk factors from the univariable analyses and inserted them in a multivariable analysis, in which we further used variable selection through Akaike's Information Criterion.

RESULTS

The BcR IG gene repertoire is more clonal in CLL-type MBL versus low-count CLL-type MBL

More abundant BcR IGH and BcR IGK/L clonotypes were present in low-count CLL-type MBL (8 and 7.2, respectively) versus CLL-type MBL cases (3.4 and 3, respectively). Yet, their cumulative frequency was higher in CLL-type MBL (medians: 93.4% and 98.3%, respectively) versus low-count CLL-type MBL (median values: 92.1% and 94.1%, respectively). Stereotypy analysis of abundant BcR IGH clonotypes (individual frequency of >0.92%) showed that a significant fraction (80/223, 35.9%) were assigned to CLL-specific, stereotyped subsets (**Supplementary Table 3)**. Abundant clonotypes corresponding to CLL-specific stereotyped BcR IG were identified in 11/14 (78.6%) CLL-type MBL samples and 18/22 (81.8%) low-count CLL-type MBL samples. The size of such stereotyped BcR IGH clonotypes was significantly higher (p<0.001) in CLL-type MBL compared to low-count CLL-like MBL (mean values: 40.85% versus 13.85%, respectively).

Distinct BcR IGH gene repertoire restrictions in low-count CLL-type MBL, CLL-type MBL and CLL

Three IGHV genes (namely IGHV3-7, IGHV3-23 and IGHV4-34) were identified as frequent (frequency>5%) in both MBL subtypes as well as CLL, while IGHV3-30 was frequent in low-count CLL-type MBL and CLL. On the other hand, the IGHV3-33 and IGHV5-51 genes were frequent exclusively in low-count CLL-type MBL, whereas IGHV3-15, IGHV3-21, IGHV3-53, IGHV3-9 and IGHV4-4 were frequent only in CLL-type MBL. Finally, a high frequency of the IGHV1-69 gene was restricted to CLL, while being virtually absent in both MBL subtypes.

Turning to the light chains, the IGKV1-39/1D-39 gene (utilized in aggressive CLL stereotyped subsets #1 and #8) was more frequent in CLL and CLL-type MBL versus low-count CLL-type MBL (p<0.05 for the comparison between CLL and low-count CLL-like MBL). In contrast, the IGLV3-21 gene (utilized in

aggressive CLL stereotyped subset #2) was frequent only in CLL, whereas it was identified in a single CLL-type MBL case ($p<0.05$ for the comparison between CLL and low-count CLL-like MBL).

BcR IG gene rearrangements in MBL exhibit higher SHM levels compared to CLL

The distribution of BcR IGH clonotypes among t3 mutational subgroups [i.e. mutated, germline identity (GI) <98%; unmutated, 98% ≤ GI <99.9%; truly unmutated, GI = 100%] differed significantly ($p<0.001$) between low-count CLL-type MBL, CLL-type MBL and CLL. In detail, mutated BcR IGH clonotypes were overrepresented in both CLL-type MBL (85.4%) and low-count CLL-type MBL (80.6%) versus CLL (54.1%). In contrast, truly unmutated clonotypes (GI=100%) displayed a significantly ($p<0.001$) higher frequency in CLL (32.7%) compared to both MBL subtypes (low-count CLL-type MBL: 4%, CLL-type MBL: 8.3 %). Significant differences ($p<0.001$) were also evident for the BcR IGK/L clonotypes with enrichment for mutated clonotypes in low-count CLL-type MBL and CLL-type MBL (56.2% and 76.2%, respectively) versus CLL (43.4%).

Pairwise comparisons using the Benjamini-Hochberg correction revealed that the relative frequency of mutated clonotypes (GI <98%) was significantly higher ($p<0.01$) in both CLL-type MBL (85.4%) and low-count CLL-type MBL (80.6%) against CLL (54.1%). On the other hand, the relative frequency of truly unmutated clonotypes (GI=100%) was significantly lower in both subtypes of MBL, namely low-count CLL-type MBL (4%) and CLL-type MBL (8.3%), compared to CLL (32.7%) ($p<0.01$ in both comparisons).

Low-count CLL-type MBL is characterized by lower levels of intraclonal diversification compared to CLL-type MBL and CLL

Clonotypes with low/no SHM were excluded from further analysis. Overall, concordant profiles of intraclonal diversification were identified between the IG heavy and the light chain gene repertoires within each group. Focusing on the BcR IGH clonotypes, less pronounced intraclonal diversification was

seen in oligoclonal MBL versus monoclonal MBL and all CLL groups, as reflected in a higher mean value of the END metric ($p<0.05$ in all comparisons) as well as significantly lower MPL and ADe mean scores versus subset #2 and all CLL groups, respectively (**Supplementary Figure 1**). The values of all graph network metrics for each sample group are provided in **Supplementary Table 5**.

In order to expand these observations, we also assessed intraclonal diversification in a distinct cohort of 11 low-count CLL-type MBL cases from our previous study[3]. First, comparisons between the present and the previous low-count CLL-type MBL cohorts showed limited differences (**Supplementary Figure 2**). Next, we merged the clonotypes of the two cohorts and repeated the same comparisons mentioned above, once again reaching the same conclusion i.e. that low-count CLL-type MBL exhibited significantly lower intraclonal diversification complexity compared to either CLL-type MBL or CLL.

The transcriptomes of low-count CLL-type MBL and CLL-type MBL are clearly distinct from that of CLL

To validate the results of our supervised gene expression comparison between MBL subtypes and CLL, we also performed an unsupervised analysis using only the significantly expressed genes (logFC >2) and the established Euclidean distance; three distinct clusters were formed, evident in the heatmap depicted in **Supplementary Figure 1B**. Cluster 1 comprised all 74 CLL cases along with 2 CLL-type MBL cases, while clusters 2 and 3 comprised exclusively MBL cases, with low-count CLL-type MBL cases representing the majority in cluster 2 (11/13 cases). Cluster 3, included 4 CLL-type MBL and 2 low-count CLL-type MBL cases. Gene enrichment analysis (**Supplementary Table 7)** revealed an upregulation ($p<0.05$) of the Notch and p53 signaling pathways in cluster 1. Clusters 2 and 3 displayed upregulation ($p<0.05$) of the antigen processing and presentation process along with a gene expression signature implicated in primary immunodeficiency.

BcR IG in MBL display broad autoantigen reactivity profiles albeit weak antigen interactions

Reactivity of rmAbs from MBL and CLL was tested against 123 autoantigens and the results were used for unsupervised clustering, leading to the formation of 3 clusters (**Supplementary Figure 3**). Cluster 1 consisted of all subset #4 cases and 1 low-count CLL-type MBL case: the respective rmAbs reacted strongly only against proteins implicated in interactions between the extracellular matrix and cell receptors, as well as small nuclear ribonucleoproteins. Cluster 2 comprised all subset #1 cases, as well as 1 CLL-type MBL and 1 low-count CLL-type MBL case: the respective rmAbs displayed strong reactivity against almost all autoantigen proteins involved in the complement and coagulation cascades. Finally, cluster 3 included most CLL-type MBL and low-count CLL-type MBL cases, all cases of CLL subsets #2/169 and the non-stereotyped CLL cases; this cluster was characterized by weak recognition against autoantigens from both groups and appeared to consist of 2 subclusters, one of which comprised almost exclusively MBL (7/8 cases). BcR IG reactivity profiling based on the protein microarray was validated by ELISA against selected specific autoantigens (data not shown). The output of principal component analysis (PCA), depicted in **Supplementary Figure 4**, corroborated the clustering findings given the formation of 3 clusters, the 2 consisting of subset #1 and #4 cases, respectively, and one containing most MBL and non-stereotyped CLL cases.

Reactivity of 15 rmAbs from 6 low-count CLL-type MBL and 9 CLL-type MBL cases was also tested against a series of pathogens. According to the reactivity patterns, depicted in **Supplementary Figure 5**, none of the MBL rmAbs was reactive against EBV and *Helicobacter pylori*, while strong reactivity against CMV was noted in 2 low-count CLL-type MBL cases. Furthermore, *Mycoplasma pneumoniae* and Influenza A virus were recognized by 4 and 3 low-count CLL-type MBL rmAbs,

respectively. Among the reactive rmAbs, those from cases LC-MBL_8 and LC-MBL_14 exhibited a polyreactive pattern recognizing all 3 aforementioned pathogens.

REFERENCES

1 Fazi C, Scarfò L, Pecciarini L, Cottini F, Dagklis A, Janus A *et al.* General population low-count CLL-like MBL persists over time without clinical progression, although carrying the same cytogenetic abnormalities of CLL. *Blood* 2011; **118**: 6618–6625.

2 Agathangelidis A, Ljungström V, Scarfò L, Fazi C, Gounari M, Pandzic T *et al.* Highly similar genomic landscapes in monoclonal B-cell lymphocytosis and ultra-stable chronic lymphocytic leukemia with low frequency of driver mutations. *Haematologica* 2018; **103**: 865–873.

3 Agathangelidis A, Galigalidou C, Scarfò L, Moysiadis T, Rovida A, Gounari M *et al.* Infrequent “chronic lymphocytic leukemia-specific” immunoglobulin stereotypes in aged individuals with or without low-count monoclonal B-cell lymphocytosis. *Haematologica* 2020; **106**: 1178–1181.

4 Alamyar E, Duroux P, Lefranc M-P, Giudicelli V. IMGT® Tools for the Nucleotide Analysis of Immunoglobulin (IG) and T Cell Receptor (TR) V-(D)-J Repertoires, Polymorphisms, and IG Mutations: IMGT/V-QUEST and IMGT/HighV-QUEST for NGS. In: *Methods in molecular biology (Clifton, N.J.)*. 2012, pp 569–604.

5 Kotouza MT, Gemenetzi K, Galigalidou C, Vlachonikola E, Pechlivanis N, Agathangelidis A *et al.* TRIP - T cell receptor/immunoglobulin profiler. *BMC Bioinformatics* 2020; **21**: 422.

6 Sofou E, Vlachonikola E, Zaragoza-Infante L, Brüggemann M, Darzentas N, Groenen PJTA *et al.* Clonotype definitions for immunogenetic studies: proposals from the EuroClonality NGS Working Group. *Leukemia* 2023; **37**: 1750–1752.

7 Zaragoza-Infante L, Junet V, Pechlivanis N, Fragkouli S-C, Amprachamian S, Koletsa T *et al.* IgIDivA: immunoglobulin intraclonal diversification analysis. *Brief Bioinform* 2022; **23**. doi:10.1093/bib/bbac349.

8 Agathangelidis A, Chatzidimitriou A, Gemenetzi K, Giudicelli V, Karypidou M, Plevova K *et al.* Higher-order connections between stereotyped subsets: implications for improved patient classification in CLL. *Blood* 2021; **137**: 1365–1376.

9 Smedby KE, Wästerlid T, Tham E, Haider Z, Joelsson J, Thorvaldsdottir B *et al.* The BioLymph study – implementing precision medicine approaches in lymphoma diagnostics, treatment and follow-up: feasibility and first results. *Acta Oncol (Madr)* 2023; **62**: 560–564.

10 Haider Z, Wästerlid T, Spångberg LD, Rabbani L, Jylhä C, Thorvaldsdottir B *et al.* Whole-genome informed circulating tumor DNA analysis by multiplex digital PCR for disease monitoring in B-cell lymphomas: a proof-of-concept study. *Front Oncol* 2023; **13**. doi:10.3389/fonc.2023.1176698.

11 Li H, Handsaker B, Wysoker A, Fennell T, Ruan J, Homer N *et al.* The Sequence Alignment/Map format and SAMtools. *Bioinformatics* 2009; **25**: 2078–2079.

12 Lai Z, Markovets A, Ahdesmaki M, Chapman B, Hofmann O, McEwen R *et al.* VarDict: a novel and versatile variant caller for next-generation sequencing in cancer research. *Nucleic Acids Res* 2016; **44**: e108–e108.

13 Landau DA, Tausch E, Taylor-Weiner AN, Stewart C, Reiter JG, Bahlo J *et al.* Mutations driving CLL and their evolution in progression and relapse. *Nature* 2015; **526**: 525–530.

14 Puente XS, Beà S, Valdés-Mas R, Villamor N, Gutiérrez-Abril J, Martín-Subero JI *et al.* Non-coding recurrent mutations in chronic lymphocytic leukaemia. *Nature* 2015; **526**: 519–524.

15 Robbe P, Ridout KE, Vavoulis D V., Dréau H, Kinnersley B, Denny N *et al.* Whole-genome sequencing of chronic lymphocytic leukemia identifies subgroups with distinct biological and clinical features. *Nat Genet* 2022; **54**: 1675–1689.

16 McLaren W, Gil L, Hunt SE, Riat HS, Ritchie GRS, Thormann A *et al.* The Ensembl Variant Effect Predictor. *Genome Biol* 2016; **17**: 122.

17 Ferreira PG, Jares P, Rico D, Gómez-López G, Martínez-Trillos A, Villamor N *et al.* Transcriptome characterization by RNA sequencing identifies a major molecular and clinical subdivision in chronic lymphocytic leukemia. *Genome Res* 2014; **24**: 212–226.

18 Kim D, Paggi JM, Park C, Bennett C, Salzberg SL. Graph-based genome alignment and genotyping with HISAT2 and HISAT-genotype. *Nat Biotechnol* 2019; **37**: 907–915.

19 Ritchie ME, Phipson B, Wu D, Hu Y, Law CW, Shi W *et al.* limma powers differential expression analyses for RNA-sequencing and microarray studies. *Nucleic Acids Res* 2015; **43**: e47–e47.

20 Chen EY, Tan CM, Kou Y, Duan Q, Wang Z, Meirelles GV *et al.* Enrichr: interactive and collaborative HTML5 gene list enrichment analysis tool. *BMC Bioinformatics* 2013; **14**: 128.

21 Goldstein LD, Cao Y, Pau G, Lawrence M, Wu TD, Seshagiri S *et al.* Prediction and Quantification of Splice Events from RNA-Seq Data. *PLoS One* 2016; **11**: e0156132.

22 Iatrou A, Gounari M, Sofou E, Zaragoza-Infante L, Markopoulos I, Sarrigeorgiou I *et al.* N-Glycosylation of the Ig Receptors Shapes the Antigen Reactivity in Chronic Lymphocytic Leukemia Subset #201. *The Journal of Immunology* 2023; **211**: 743–754.

23 Maza E, Frasse P, Senin P, Bouzayen M, Zouine M. Comparison of normalization methods for differential gene expression analysis in RNA-Seq experiments. *Commun Integr Biol* 2013; **6**: e25849.

24 Dighiero G, Lymberi P, Holmberg D, Lundquist I, Coutinho A, Avrameas S. High frequency of natural autoantibodies in normal newborn mice. *J Immunol* 1985; **134**: 765–71.

25 Hatzioannou A, Kanistras I, Mantzou E, Anastasiou E, Peppa M, Sarantopoulou V *et al.* Effect of Advanced Glycation End Products on Human Thyroglobulin's Antigenicity as Identified by the Use of Sera from Patients with Hashimoto's Thyroiditis and Gestational Diabetes Mellitus. *Int J Endocrinol* 2015; **2015**: 1–7.

26 Sali AD, Karakasiliotis I, Evangelidou M, Avrameas S, Lymberi P. Immunological evidence and regulatory potential for cell-penetrating antibodies in intravenous immunoglobulin. *Clin Transl Immunology* 2015; **4**: e42.

27 Maity PC, Bilal M, Koning MT, Young M, van Bergen CAM, Renna V *et al.* *IGLV3-21 * 01* is an inherited risk factor for CLL through the acquisition of a single-point mutation enabling

autonomous BCR signaling. *Proceedings of the National Academy of Sciences* 2020; **117**: 4320–4327.

28 Nicolò A, Linder AT, Jumaa H, Maity PC. The Determinants of B Cell Receptor Signaling as Prototype Molecular Biomarkers of Leukemia. *Front Oncol* 2021; **11**. doi:10.3389/fonc.2021.771669.

SUPPLEMENTARY FIGURES

Supplementary Figure 1. Less pronounced intraclonal in oligoclonal MBL compared to CLL and monoclonal MBL. Oligoclonal MBL was found to exhibit lower intraclonal diversification complexity, as evidenced by the significantly higher mean value of the END metric versus all other groups, as well as significantly lower mean values of the MPL (versus subset #2) and Ade (versus all CLL groups). The only exception was the RRC metric, where oligoclonal MBL exhibited a significantly higher value versus subset #4 and non-subset CLL.

Box plot demonstrating the distribution of the metrics for the BcR IGH clonotypes in all sample groups. The x-axis represents the sample groups, namely CLL stereotyped subset #2, CLL stereotyped subset #4, non-subset CLL, monoclonal MBL and oligoclonal MBL, while the y-axis represents the metrics values. Convergence score: RRC; end nodes density: END; max path length: maximal pathway length, MPL; max muts length: maximal mutational length, MML; avg degree: average degree, ADe; avg distance: average distance, Adi. Statistical analysis concerned the application of the Kruskal-Wallis test, which was applied to determine whether the comparisons were overall statistically significant. Next, pairwise comparisons were performed for all sample groups and the Wilcoxon test was performed to determine their statistical significance. Statistically significant differences among sample groups are depicted through horizontal bars connecting the two groups along with the corresponding p-value. If the overall comparison was statistically significant, the p-value obtained from the Kruskal-Wallis test is shown at the bottom right corner of the graph.

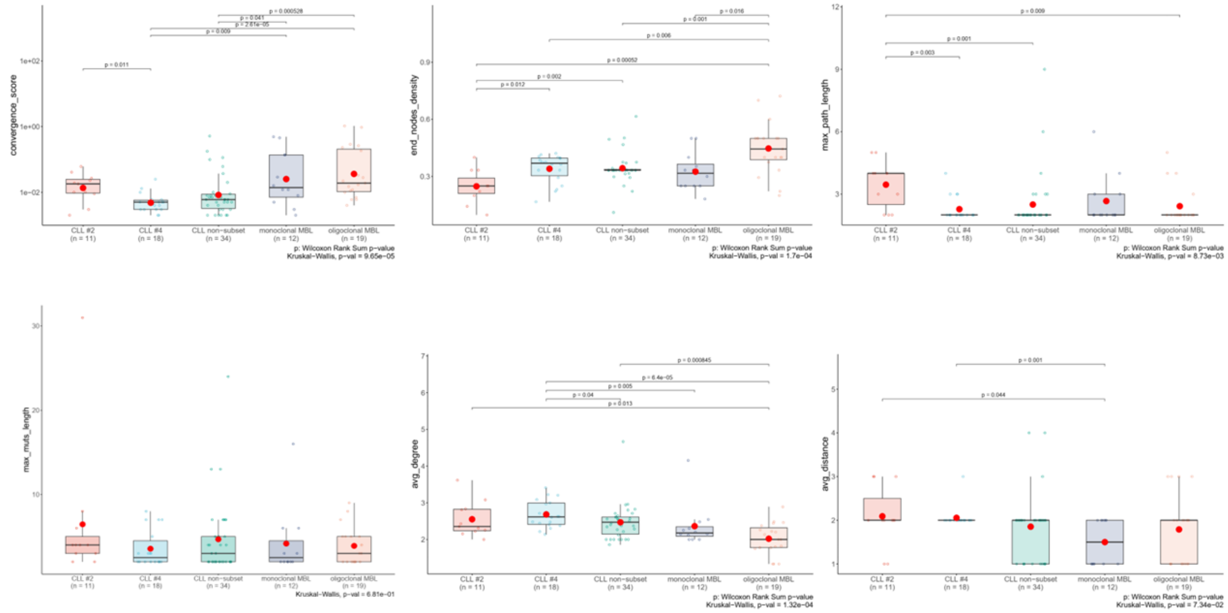
convergence_score
end_nodes_density
max_path_length
max_muts_length
avg_degree
avg_distance
CLL #2 (n = 11)
CLL #4 (n = 18)
CLL non−subset (n = 34)
monoclonal MBL (n = 12)
oligoclonal MBL (n = 19)
p: Wilcoxon Rank Sum p-value

Supplementary Figure 2. Heatmaps of supervised (A) and unsupervised (B) hierarchical clustering based on RNA-seq data of low-count CLL-type MBL, CLL-type MBL and CLL samples. Columns represent samples and rows represent genes, respectively. Sample origin is depicted through a color code (green: low-count CLL-type MBL, red: CLL-type MBL and blue: CLL). The color bar indicates mRNA expression levels (dark red indicates high expression; dark blue indicates low expression).

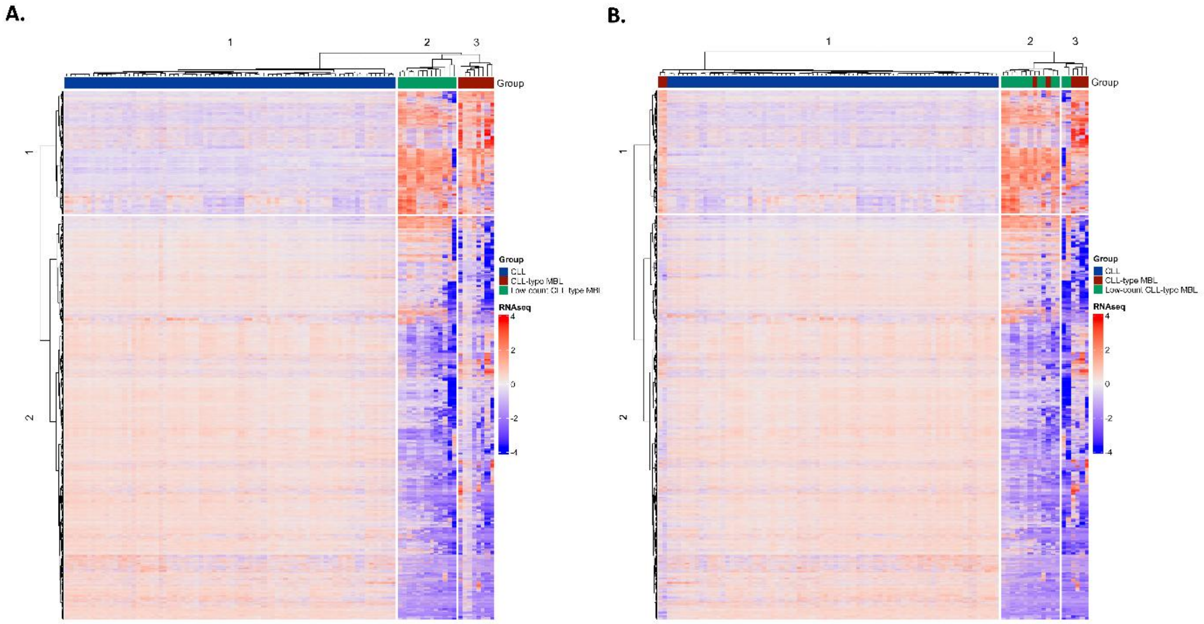

Supplementary Figure 3. BcR IG reactivity analysis against various autoantigens revealed a polyreactive profile for most MBL cases. Analysis of autoantibody profiling of MBL and CLL rmAbs (n=28). MBL and CLL rmAbs were used as primary antibodies. Each row of the heatmap corresponds to a different antigen while every column represents a sample. Heatmap is depicted with dark red representing the highest relative reactivity level and dark purple representing the absence of relative antibody activity level, as indicated in the legend.

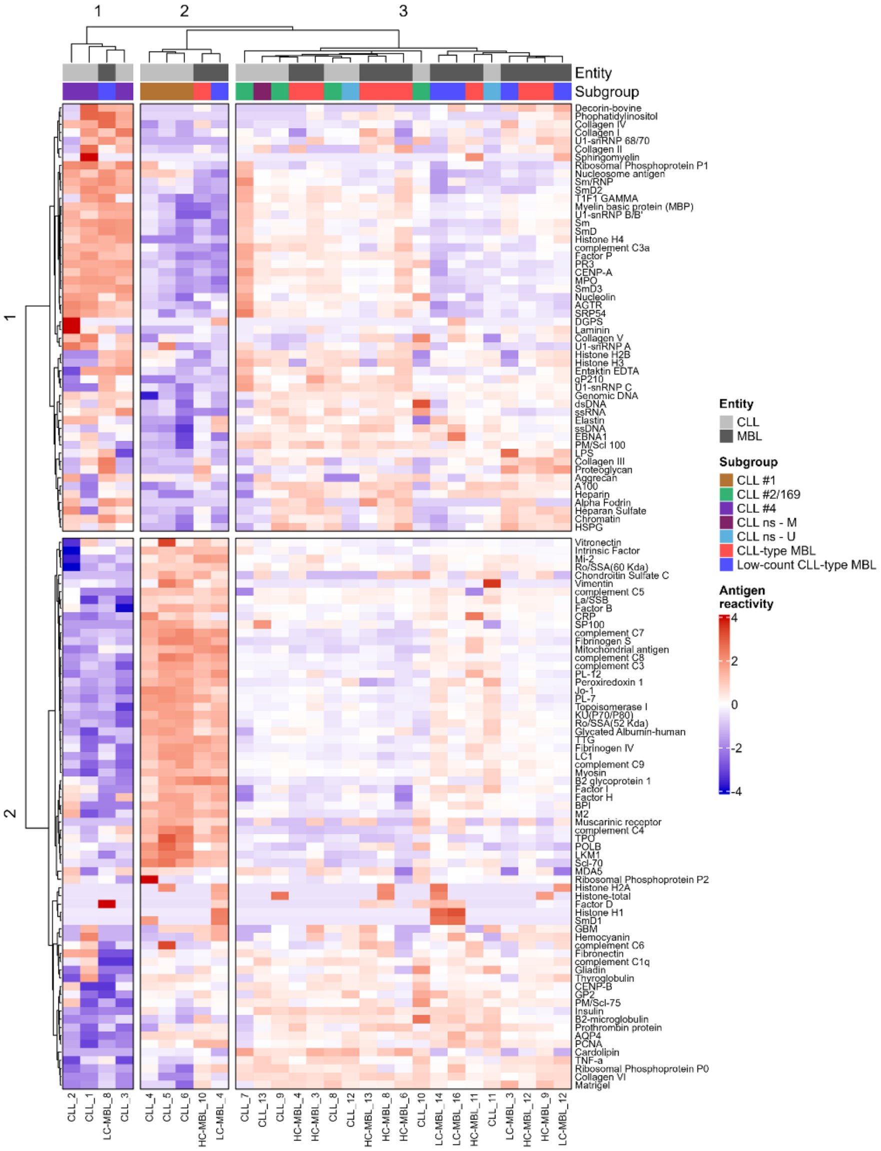

Supplementary Figure 4. Variance of antigen reactivity assessed by principal component analysis. The majority of MBL cases form a group with some CLL cases, mostly expressing heterogeneous BcR IG. Of interest, CLL cases from subsets #1 and #4 formed distinct clusters, indicating the unique reactivity patterns of BcR IG belonging to stereotyped subsets. Finally, 1 CLL-type MBL and 2 low-count CLL-type MBL cases, previously assigned to clusters 1 and 2, are found in the space between the aforementioned groups.

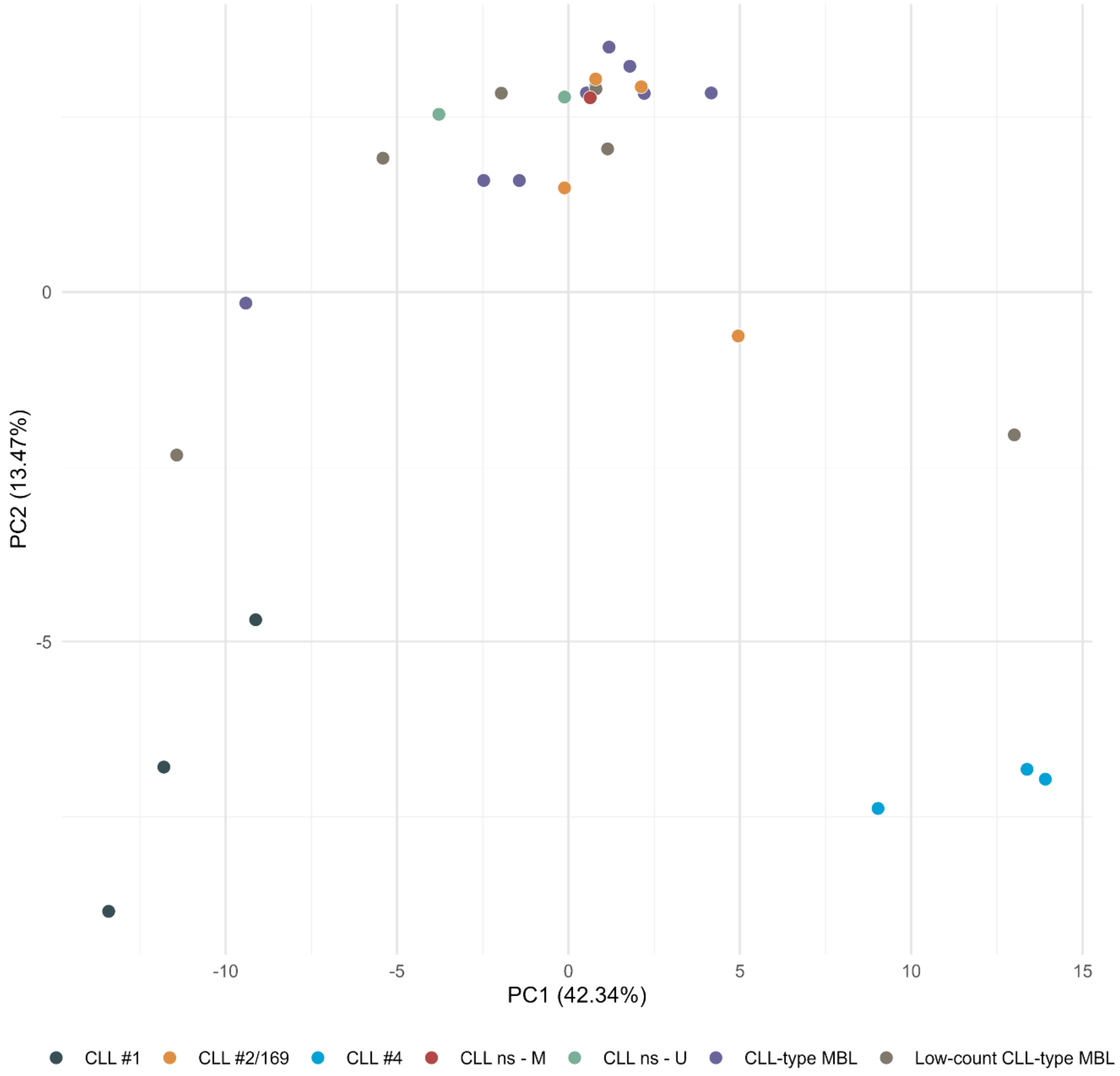

Supplementary Figure 5. ELISA against microbes. Reactivity against (A) Cytomegalovirus (CMV), (B) Influenza A virus, (C) Epstein-Barr virus (EBV), (D) Helicobacter pylori and (E) Mycoplasma pneumoniae was tested by commercial ELISA kits. In all ELISAs MBL and CLL rmAbs were used in duplicates as primary antibodies. Each bar corresponds to the mean OD value. The cut-off value of the weak positivity is indicated by dotted horizontal lines. Identifiers for low-count CLL-type MBL and CLL-type MBL cases are abbreviated as LC-MBL and HC-MBL, respectively.

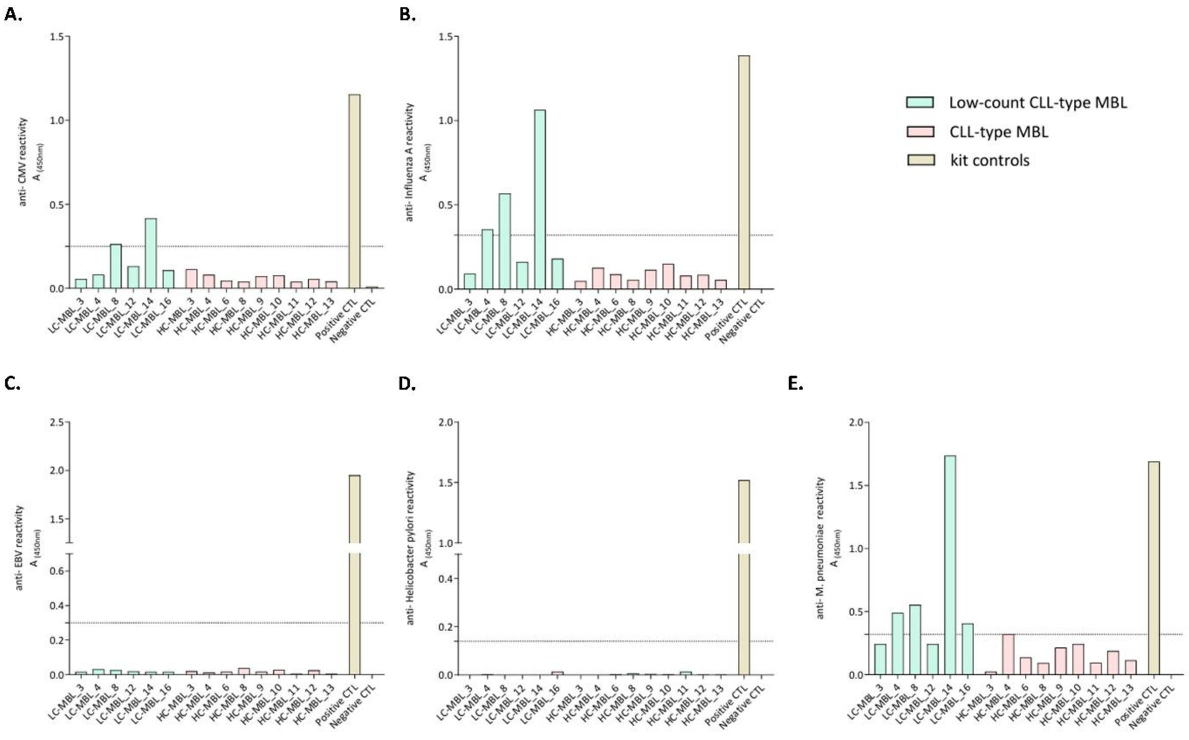

Supplement: Supplementary file 1 — Supplementary material [file 41375_2025_2797_MOESM1_ESM.pdf]
