# Supplementary material for: Molecular and functional characterization of the B-cell receptor in chronic lymphocytic leukemia-like monoclonal B-cell lymphocytosis
Source: Leukemia. 2026 Jan 7;40(2):454–8. doi: 10.1038/s41375-025-02797-y (PMC12875869; doi:10.1038/s41375-025-02797-y)
Supplement: Supplementary file 2 — Supplementary tables [file 41375_2025_2797_MOESM2_ESM.pdf]

Supplementary Table 1. Basic information regarding all individual cases of the present MBL cohort.

Sample ID	Entity	Gender	Age	Light chain isotype	CLL clone, cells/µl	Cell purity, %
HC-MBL_1	CLL-type MBL	M	84	Kappa	4986.2	96.8
HC-MBL_2	CLL-type MBL	M	86	Lambda	1379.4	99.2
HC-MBL_3	CLL-type MBL	M	80	Kappa	1202.6	99
HC-MBL_4	CLL-type MBL	M	72	Kappa	1168.9	98.4
HC-MBL_5	CLL-type MBL	F	71	Lambda	1229.7	99.2
HC-MBL_6	CLL-type MBL	F	85	Kappa	4796.8	96.9
HC-MBL_7	CLL-type MBL	M	76	Kappa	626.4	96.9
HC-MBL_8	CLL-type MBL	M	86	Kappa	1029.5	98.5
HC-MBL_9	CLL-type MBL	F	85	Kappa	1939.1	97.9
HC-MBL_10	CLL-type MBL	M	74	Kappa	512.5	98.9
HC-MBL_11	CLL-type MBL	M	72	Kappa	2156.7	97.3
HC-MBL_12	CLL-type MBL	F	79	Kappa	3561.8	98.6
HC-MBL_13	CLL-type MBL	M	84	Kappa	4197.4	98.4
HC-MBL_14	CLL-type MBL	M	84	Lambda	2487	98.9
LC-MBL_1	Low-count CLL-type MBL	M	92	Kappa	6.6	98.3
LC-MBL_2	Low-count CLL-type MBL	M	75	Lambda	48.4	98.7
LC-MBL_3	Low-count CLL-type MBL	F	79	Kappa	2.9	99.1
LC-MBL_4	Low-count CLL-type MBL	M	97	Kappa	189.8	97.3
LC-MBL_5	Low-count CLL-type MBL	F	81	Kappa	6.5	97.3
LC-MBL_6	Low-count CLL-type MBL	M	77	Kappa	64.2	97.1
LC-MBL_7	Low-count CLL-type MBL	M	71	Lambda	1.4	97.7
LC-MBL_8	Low-count CLL-type MBL	M	88	Kappa	95.5	99
LC-MBL_9	Low-count CLL-type MBL	F	74	Kappa/Lambda	0.4	98.3
LC-MBL_10	Low-count CLL-type MBL	M	54	Kappa	0.6	98.5
LC-MBL_11	Low-count CLL-type MBL	M	62	Kappa	3	96.8
LC-MBL_12	Low-count CLL-type MBL	M	74	Kappa	55.5	98.1
LC-MBL_13	Low-count CLL-type MBL	M	81	Kappa	0.1	97.4
LC-MBL_14	Low-count CLL-type MBL	F	79	Kappa	150	99.1
LC-MBL_15	Low-count CLL-type MBL	M	73	Kappa	0.9	96.9
LC-MBL_16	Low-count CLL-type MBL	M	62	Lambda	343	98.6
LC-MBL_17	Low-count CLL-type MBL	F	56	Kappa	11	96.2
LC-MBL_18	Low-count CLL-type MBL	M	85	Kappa	21	97.4
LC-MBL_19	Low-count CLL-type MBL	M	60	Kappa	5.3	98.6
LC-MBL_20	Low-count CLL-type MBL	M	64	Unknown	6.6	96.9
LC-MBL_21	Low-count CLL-type MBL	M	80	Kappa	2.7	99.2
LC-MBL_22	Low-count CLL-type MBL	M	73	Kappa	0.6	98.4

Supplementary Table 2. Clonality levels in the BcR IGH and BcR IGK/L repertoires in each sample of the present MBL cohort.

			Heavy chain					Light chain				
Entity	Sample ID	Clonality level	Clonotype ID	IGHV gene	CDR3	N	Freq	Clonotype ID	Gene	CDR3	N	Freq
CLL-type MBL	HC-MBL_1	biclonal	HC-MBL_1.1_H	IGHV4-31	CARARAYDTNDYYPVKFDSW	66348	55.76	HC-MBL_1.1_K	IGKV2-28	CMQVLQTPLTF	117496	92.73
				IGHV3-15	CVTDDFGDRVTGTPVDFFDSW	42567	35.77	HC-MBL_1.2_K	IGKV3-15	CQQFNYWPGTF	5101	4.03
CLL-type MBL	HC-MBL_2	monoclonal	HC-MBL_2.1_H	IGHV3-9	CAKDIFLYSGSYYHAFDIW	85517	94.15	HC-MBL_2.1_L	IGLV2-14	CNSYTGTSTLVVF	175668	98.61
CLL-type MBL	HC-MBL_3	monoclonal	HC-MBL_3.1_H	IGHV3-30	CAKEGSASKWSFDLW	114410	98.24	HC-MBL_3.1_K	IGKV3-11	CQQRGDWPPDATF	105558	88.07
CLL-type MBL	HC-MBL_4	monoclonal	HC-MBL_4.1_H	IGHV3-48	CARGRDGYNRCPFDYW	46542	50.70	HC-MBL_4.1_K	IGKV1-8	CQQYYTFPGAF	117723	99.44
			HC-MBL_4.2_H	IGHV4-34	CARRVQPGDNYYFGMDVW	42801	46.62	-	-	–	-	-
CLL-type MBL	HC-MBL_5	monoclonal	HC-MBL_5.1_H	IGHV3-21	CAGDRNGMDVW	49642	48.51	HC-MBL_5.1_L	IGLV3-21	CQVWDGSSDHPWVF	103503	79.91
CLL-type MBL	HC-MBL_6	monoclonal	HC-MBL_6.1_H	IGHV3-15	CSRQAHIDFQYW	90080	86.51	HC-MBL_6.1_K	IGKV3-20	CQQYDLVPYTF	149843	89.78
CLL-type MBL	HC-MBL_7	oligoclonal	HC-MBL_7.1_H	IGHV4-4	CARSRDRSGYQEEGFDYW	7246	26.58	HC-MBL_7.1_K	IGKV1-33	CQHYENLPYTF	22549	88.66
			HC-MBL_7.2_H	IGHV3-21	CARDLIAAAPGAFDIW	5481	20.11	HC-MBL_7.2_K	IGKV2-24	CMQATQFPWTF	1173	4.61
			HC-MBL_7.3_H	IGHV1-8	CARGLGAGPAADNRDIW	2657	9.75	HC-MBL_7.3_K	IGKV1-13	CQQFNSFPLTF	986	3.88
CLL-type MBL	HC-MBL_8	monoclonal	HC-MBL_8.1_H	IGHV3-20	CARDPMGNYDILTGYFVDAFDIW	80717	94.14	HC-MBL_8.1_K	IGKV3-20	CQHYGNSPYTF	89512	88.76
CLL-type MBL	HC-MBL_9	monoclonal	HC-MBL_9.1_H	IGHV4-34	CVRGYGVESTLRRYYYYGMDVW	66785	96.03	HC-MBL_9.1_K	IGKV2-30	CMQGTHWPPYTF	467	92.84
CLL-type MBL	HC-MBL_10	monoclonal	HC-MBL_10.1_H	IGHV4-34	CAREKDSGGNSLLWYW	29861	86.05	HC-MBL_10.1_K	IGKV1-8	CLQYYSYPRTF	142294	99.86
CLL-type MBL	HC-MBL_11	monoclonal	HC-MBL_11.1_H	IGHV5-51	CARGGNYVDIW	72729	88.11	HC-MBL_11.1_K	IGKV3-15	CQQYNNWPRTF	222094	99.92
CLL-type MBL	HC-MBL_12	monoclonal	HC-MBL_12.1_H	IGHV4-34	CARLKGYGSGTGSYNYFDYW	45252	98.15	HC-MBL_12.1_K	IGKV4-1	CQQYYSTPRTF	6131	19.33
CLL-type MBL	HC-MBL_13	monoclonal	HC-MBL_13.1_H	IGHV1-69	CAREGKSTVAGPIDYW	40259	78.08	HC-MBL_13.1_K	IGKV3-20	CQQYGSSPMTF	147448	99.92
CLL-type MBL	HC-MBL_14	monoclonal	HC-MBL_14.1_H	IGHV4-4	CARHLDTSGFYSDYW	13093	30.32	HC-MBL_14.1_L	IGLV1-44	CTAWDDSLNSWMF	194961	99.66
			HC-MBL_14.2_H		CARHVDSSGYYSDYW	12727	29.47	-	-	–	-	-
Low-count CLL-type MBL	LC-MBL_1	oligoclonal	LC-MBL_1.1_H	IGHV3-7	CAVAPYSSSSSQRYW	34947	36.66	LC-MBL_1.1_K	IGKV1-5	CQQYNSYPLTF	40210	57.45
			LC-MBL_1.2_H	IGHV3-7	CATPPYCDTSCPATDVW	24904	26.12	LC-MBL_1.2_K	IGKV2-28	CMQALQTPITF	14601	20.86
			LC-MBL_1.3_H	IGHV4-34	CARGGYSVTSLKYGMDVW	22415	23.51	LC-MBL_1.3_K	IGKV1-12	CQQANSFPLTF	6117	8.74
Low-count CLL-type MBL	LC-MBL_2	monoclonal	LC-MBL_2.1_H	IGHV4-34	CVRNMPNSHTPGWFDPW	106470	87.91	LC-MBL_2.1_L	IGLV2-14	CCSYTGSGTLYVF	225798	91.33
Low-count CLL-type MBL	LC-MBL_3	monoclonal	LC-MBL_3.1_H	IGHV3-7	CARGPGYLIDFW	52167	96.04	LC-MBL_3.1_K	IGKV4-1	CLQIHSSPVTF	37524	93.31
Low-count CLL-type MBL	LC-MBL_4	monoclonal	LC-MBL_4.1_H	IGHV1-18	CARGTRVTTSPYYYYMDVW	56756	78.54	LC-MBL_4.1_K	IGKV2-28	CMQALQTPWTF	220992	99.53

Low-count CLL-type MBL	LC-MBL_5	oligoclonal	LC-MBL_5.1_H	IGHV1-3	CARGIRVGYDYGYDVFDIW	4177	31.28	LC-MBL_5.1_K	IGKV1D-12	CQQANSYPFSF	17635	84.94
			LC-MBL_5.2_H	IGHV4-59	CARVANAVAGLGYFDYW	2449	18.34	-	-	–	-	-
			LC-MBL_5.3_H	IGHV4-39	CVFRQSGYYLATW	1289	9.65	-	-	–	-	-
			LC-MBL_5.4_H	IGHV3-48	CARDHSSSSPAGDYW	1126	8.43	-	-	–	-	-
Low-count CLL-type MBL	LC-MBL_6	oligoclonal	LC-MBL_6.1_H	IGHV4-34	CARRGGVDYW	30938	29.32	LC-MBL_6.1_K	IGKV2-30	CMQGTHWPPPF	9606	40.52
			LC-MBL_6.2_H	IGHV4-34	CARGGEQWLGFAFDFW	18908	17.92	LC-MBL_6.2_K	IGKV1-5	CQQYGSYSGVTF	5196	21.92
			LC-MBL_6.3_H	IGHV3-72	CVRVGDYFDSSGSSLDALDFW	16410	15.55	LC-MBL_6.3_K	IGKV1-5	CQQYSSYSQTF	1623	6.85
Low-count CLL-type MBL	LC-MBL_7	monoclonal	LC-MBL_7.1_H	IGHV3-53	CASEVAGNIDYGMDVW	61604	84.66	LC-MBL_7.1_L	IGLV2-8	CSSYAGSNNVF	46316	46.04
Low-count CLL-type MBL	LC-MBL_8	biclonal	LC-MBL_8.1_H	IGHV3-15	CSTDRPFSGATTFDYW	35595	43.80	LC-MBL_8.1_K	IGKV3-20	CQQYERPPWTF	35784	35.49
			LC-MBL_8.2_H	IGHV3-43	CAKELGRRSWSEVDSW	25152	30.95	LC-MBL_8.2_K	IGKV3-15	CQQYKNWPLTF	22493	22.31
Low-count CLL-type MBL	LC-MBL_9	oligoclonal	LC-MBL_9.1_H	IGHV3-23	CAKGAVTGMGAPFDYW	22963	29.44	LC-MBL_9.1_K	IGKV1-39	CQQSYSTPRTF	32753	35.26
			LC-MBL_9.2_H	IGHV3-21	CARDGDAFDIW	3914	5.02	LC-MBL_9.2_L	IGLV2-14	CSSYTSSSTVVF	62944	32.63
Low-count CLL-type MBL	LC-MBL_10	oligoclonal	LC-MBL_10.1_H	IGHV3-33	CTNFDYW	12464	10.71	LC-MBL_10.1_K	IGKV2-28	CMQTLQTPNSF	25794	18.25
			LC-MBL_10.2_H	IGHV1-8	CARAPRRAGIIIKDWFDPW	7601	6.53	LC-MBL_10.2_K	IGKV1-27	CQKYDTARRTF	8087	5.72
			LC-MBL_10.3_H	IGHV3-30	CYYDTHSDYW	6121	5.26	LC-MBL_10.3_K	IGKV4-1	CQHYYTTPWTF	6473	4.58
			LC-MBL_10.4_H	IGHV3-30	CAKDSIPMDVW	5801	4.99	LC-MBL_10.4_K	IGKV4-1	CQQYYSSPITF	6120	4.33
Low-count CLL-type MBL	LC-MBL_11	oligoclonal	LC-MBL_11.1_H	IGHV4-34	CASTRFDILLWFGDLPTTSIHFGSW	14281	14.97	LC-MBL_11.1_K	IGKV3-20	CQQYGSSPSF	10159	15.19
			LC-MBL_11.2_H	IGHV3-53	CVRERIEGGEPAPIRSGAFDIW	14107	14.79	LC-MBL_11.2_K	IGKV4-1	CQQYHHILWTF	7351	10.99
			LC-MBL_11.3_H	IGHV3-53	CTRDGRGESSGYRGFDYW	5436	5.70	LC-MBL_11.3_K	IGKV3-11	CQQRSNWPTF	4963	7.42
Low-count CLL-type MBL	LC-MBL_12	monoclonal	LC-MBL_12.1_H	IGHV1-8	CARGLADNTDYW	47638	75.72	LC-MBL_12.1_K	IGKV3-20	CQQYGRSPNTF	128648	95.13
Low-count CLL-type MBL	LC-MBL_13	oligoclonal	LC-MBL_13.1_H	IGHV3-33	CARDPQGDIVATIYGYGYYYGMDVW	1272	10.65	LC-MBL_13.1_K	IGKV1-5	CHQYDSYLHSF	8882	20.65
			LC-MBL_13.2_H	IGHV2-5	CVHYNQWGSHFDYW	576	4.82	LC-MBL_13.2_K	IGKV1-5	CQQYNGPWTF	5443	12.65
Low-count CLL-type MBL	LC-MBL_14	monoclonal	LC-MBL_14.1_H	IGHV1-2	CARDTSGSCVYW	50797	69.73	LC-MBL_14.1_K	IGKV2-28	CMQALQTPRTF	186963	99.16
Low-count CLL-type MBL	LC-MBL_15	oligoclonal	LC-MBL_15.1_H	IGHV3-7	CARDGWDNLYDYW	5051	4.85	LC-MBL_15.1_K	IGKV4-1	CQQYYSTPYSF	5597	11.43
			LC-MBL_15.2_H	IGHV3-15	CTGDIVGGTTSFDYW	4748	4.56	LC-MBL_15.2_K	IGKV2-30	CMQGTHWPYTF	3508	7.16
Low-count CLL-type MBL	LC-MBL_16	monoclonal	LC-MBL_16.1_H	IGHV5-51	CARQWVSTAITALPDYW	44145	88.45	LC-MBL_16.1_L	IGLV2-8	CGSYAGSNNFVIF	61151	98.35
Low-count CLL-type MBL	LC-MBL_17	oligoclonal	LC-MBL_17.1_H	IGHV4-34	CARLAYCGGDCYSRYYFDSW	9832	63.96	LC-MBL_17.1_K	IGKV4-1	CQQYYSIPGLTF	60753	87.24
			LC-MBL_17.2_H	IGHV3-23	CAKDRFDNSDSYYFDYW	1911	12.43	-	-	-	-	-
			LC-MBL_17.3_H	IGHV3-49	CSGYNWDYW	565	3.68	-	-	-	-	-
Low-count CLL-type MBL	LC-MBL_18	oligoclonal	LC-MBL_18.1_H	IGHV3-33	CARDPMVATNGDLLLPPGPDHW	12501	44.85	LC-MBL_18.1_K	IGKV1-5	CQQYNEYPRTF	349719	80.62
			LC-MBL_18.2_H	IGHV2-5	CAHRRGMGDFDYW	6514	23.37	-	-	-	-	-

			LC-MBL_18.3_H	IGHV3-23	CAKEFSVSGGWSPYFNFW	4570	16.40	-	-	-	-	-
Low-count CLL-type MBL	LC-MBL_19	monoclonal	LC-MBL_19.1_H	IGHV3-7	CARGDSGRAESAYW	88599	94.24	LC-MBL_19.1_K	IGKV1-5	CQQYNSYSRGF	6846	44.87
Low-count CLL-type MBL	LC-MBL_20	oligoclonal	LC-MBL_20.1_H	IGHV3-48	CARYGSGSYYRDPFDYW	6555	64.79	-	-	-	-	-
			LC-MBL_20.2_H	IGHV3-23	CARFTGQGSVPRFDQW	1360	13.44	-	-	-	-	-
			LC-MBL_20.3_H	IGHV3-11	CARYGSGSYYRDPFDYW	1157	11.44	-	-	-	-	-
Low-count CLL-type MBL	LC-MBL_21	monoclonal	LC-MBL_21.1_H	IGHV3-15	CSTEEFSGYTYGILPNYW	14306	94.47	-	-	-	-	-
Low-count CLL-type MBL	LC-MBL_22	monoclonal	LC-MBL_22.1_H	IGHV3-74	CARGIAVAGSAWYYFDYW	5969	98.27	-	-	-	-	-

Supplementary Table 3. List of abundant BcR IGH clonotypes from Low-count CLL-type MBL and CLL-type MBL assigned to "CLL-specific" stereotyped subsets.

<table>
<tr><th>Sample ID</th><th>Clonotype ID</th><th>Frequency, %</th><th>IGHV gene</th><th>IGHV germline identity %</th><th>IGHD gene</th><th>IGHD RF</th><th>IGHJ gene</th><th>CDR3 length</th><th>CDR3 amino acid sequence</th><th>Stereotyped subset</th><th>Subset size</th><th>No of subset sequences</th></tr>
<tr><td>HC-MBL_1</td><td>HC-MBL_1.3_H</td><td>3.21</td><td>IGHV4-59</td><td>93.33</td><td>IGHD2-15</td><td>2</td><td>IGHJ5</td><td>16</td><td>CARHYCSGGDCYLRFDSW</td><td>novel subset 1</td><td>minor</td><td>2</td></tr>
<tr><td>HC-MBL_2</td><td>HC-MBL_2.1_H</td><td>95.73</td><td>IGHV3-9</td><td>94.44</td><td>IGHD1-26</td><td>3</td><td>IGHJ3</td><td>17</td><td>CAKDIFLYSGSYYHAFDIW</td><td>novel subset 2</td><td>minor</td><td>3</td></tr>
<tr><td>HC-MBL_2</td><td>HC-MBL_2.2_H</td><td>2.84</td><td>IGHV3-74</td><td>92.01</td><td>IGHD2-15</td><td>3</td><td>IGHJ1</td><td>8</td><td>CLREGPDSPW</td><td>V3-7|J4.5|8|3</td><td>minor</td><td>3</td></tr>
<tr><td>HC-MBL_3</td><td>HC-MBL_3.1_H</td><td>99.90</td><td>IGHV3-30</td><td>87.85</td><td>IGHD6-13</td><td>1</td><td>IGHJ5</td><td>13</td><td>CAKEGSASKWSFDLW</td><td>V3-30|J2.4|13|6</td><td>minor</td><td>11</td></tr>
<tr><td>HC-MBL_4</td><td>HC-MBL_4.1_H</td><td>51.87</td><td>IGHV3-48</td><td>94.44</td><td>IGHD5-24</td><td>3</td><td>IGHJ4</td><td>14</td><td>CARGRDGYNRCPFDYW</td><td>76</td><td>minor</td><td>4</td></tr>
<tr><td>HC-MBL_5</td><td>HC-MBL_5.1_H</td><td>99.87</td><td>IGHV3-21</td><td>99.65</td><td>IGHD1-14</td><td>3</td><td>IGHJ6</td><td>9</td><td>CAGDRNGMDVW</td><td>2</td><td>major</td><td>698</td></tr>
<tr><td>HC-MBL_7</td><td>HC-MBL_7.2_H</td><td>22.71</td><td>IGHV3-21</td><td>99.65</td><td>IGHD6-13</td><td>2</td><td>IGHJ3</td><td>14</td><td>CARDLIAAAPGAFDIW</td><td>V3|J3|14|40</td><td>minor</td><td>9</td></tr>
<tr><td>HC-MBL_7</td><td>HC-MBL_7.4_H</td><td>9.32</td><td>IGHV3-74</td><td>94.79</td><td>IGHD3-10</td><td>2</td><td>IGHJ4</td><td>13</td><td>CSRDLIFGSGSSDYW</td><td>V3|J3.4|13|32</td><td>minor</td><td>10</td></tr>
<tr><td>HC-MBL_7</td><td>HC-MBL_7.7_H</td><td>4.06</td><td>IGHV3-9</td><td>100</td><td>IGHD3-3</td><td>2</td><td>IGHJ4</td><td>17</td><td>CAKDTSAAYDFWSGYYDYW</td><td>V3-9|J1.3.4|17|1</td><td>minor</td><td>6</td></tr>
<tr><td>HC-MBL_8</td><td>HC-MBL_8.1_H</td><td>96.57</td><td>IGHV3-20</td><td>97.57</td><td>IGHD3-9</td><td>2</td><td>IGHJ3</td><td>21</td><td>CARDPMGNYDILTGYFVDAFDIW</td><td>V3-49|J3|21|1</td><td>minor</td><td>4</td></tr>
<tr><td>HC-MBL_9</td><td>HC-MBL_9.1_H</td><td>98.16</td><td>IGHV4-34</td><td>91.23</td><td>IGHD3-3</td><td>3</td><td>IGHJ6</td><td>20</td><td>CVRGYGVESTLRRYYYYGMDVW</td><td>4</td><td>major</td><td>398</td></tr>
<tr><td>HC-MBL_10</td><td>HC-MBL_10.5_H</td><td>0.93</td><td>IGHV3-30-3</td><td>87.15</td><td>IGHD3-22</td><td>2</td><td>IGHJ3</td><td>15</td><td>CAREHSDSSGLEAFDVW</td><td>V3|J3.4|15|40</td><td>minor</td><td>48</td></tr>
<tr><td>HC-MBL_13</td><td>HC-MBL_13.1_H</td><td>78.08</td><td>IGHV1-69</td><td>93.75</td><td>IGHD6-13</td><td>2</td><td>IGHJ4</td><td>14</td><td>CAREGKSTVAGPIDYW</td><td>91</td><td>minor</td><td>20</td></tr>
<tr><td>HC-MBL_13</td><td>HC-MBL_13.2_H</td><td>1.39</td><td>IGHV3-23</td><td>92.63</td><td>IGHD2-21</td><td>3</td><td>IGHJ6</td><td>17</td><td>CAKDGVVVTAIYYYVMDVW</td><td>novel subset 3</td><td>minor</td><td>2</td></tr>
<tr><td>HC-MBL_14</td><td>HC-MBL_14.1_H</td><td>30.32</td><td>IGHV4-4</td><td>95.14</td><td>IGHD3-22</td><td>2</td><td>IGHJ4</td><td>13</td><td>CARHLDTSGFYSDYW</td><td>novel subset 4</td><td>minor</td><td>4</td></tr>
<tr><td>HC-MBL_14</td><td>HC-MBL_14.2_H</td><td>29.47</td><td>IGHV4-4</td><td>97.22</td><td>IGHD3-22</td><td>2</td><td>IGHJ4</td><td>13</td><td>CARHVDSSGYYSDYW</td><td>novel subset 4</td><td>minor</td><td>4</td></tr>
<tr><td>HC-MBL_14</td><td>HC-MBL_14.3_H</td><td>7.85</td><td>IGHV3-7</td><td>95.14</td><td>IGHD6-13</td><td>1</td><td>IGHJ4</td><td>18</td><td>CARDYRFYTTDWYRGGVDDW</td><td>novel subset 5</td><td>minor</td><td>2</td></tr>
<tr><td>HC-MBL_14</td><td>HC-MBL_14.6_H</td><td>2.48</td><td>IGHV5-51</td><td>94.44</td><td>IGHD5-18</td><td>3</td><td>IGHJ4</td><td>13</td><td>CARHRGYSYGYGDYW</td><td>V5-51|J4|13|3</td><td>minor</td><td>3</td></tr>
<tr><td>LC-MBL_1</td><td>LC-MBL_1.5_H</td><td>1.78</td><td>IGHV3-7</td><td>98.26</td><td>IGHD1-7</td><td>1</td><td>IGHJ4</td><td>7</td><td>CARGSGSIW</td><td>V3|J1.3.4.5.6|7|1</td><td>minor</td><td>9</td></tr>
<tr><td>LC-MBL_3</td><td>LC-MBL_3.1_H</td><td>96.04</td><td>IGHV3-7</td><td>91.67</td><td>IGHD2-2</td><td>3</td><td>IGHJ4</td><td>10</td><td>CARGPGYLIDFW</td><td>novel subset 6</td><td>minor</td><td>2</td></tr>
<tr><td>LC-MBL_3</td><td>LC-MBL_3.2_H</td><td>1.40</td><td>IGHV3-21</td><td>91.32</td><td>IGHD1-1</td><td>1</td><td>IGHJ4</td><td>13</td><td>CARDRGLETGNFDYW</td><td>V3|J3.4|13|2</td><td>minor</td><td>15</td></tr>
<tr><td>LC-MBL_4</td><td>LC-MBL_4.1_H</td><td>78.54</td><td>IGHV1-18</td><td>89.93</td><td>IGHD4-11</td><td>3</td><td>IGHJ6</td><td>17</td><td>CARGTRVTTSPYYYYMDVW</td><td>novel subset 7</td><td>minor</td><td>2</td></tr>
<tr><td>LC-MBL_5</td><td>LC-MBL_5.1_H</td><td>31.28</td><td>IGHV1-3</td><td>90.62</td><td>IGHD5-18</td><td>3</td><td>IGHJ3</td><td>17</td><td>CARGIRVGYDYGYDVFDIW</td><td>V1-3|J3|17|3</td><td>minor</td><td>9</td></tr>
<tr><td>LC-MBL_5</td><td>LC-MBL_5.11_H</td><td>2.01</td><td>IGHV3-21</td><td>98.61</td><td>IGHD2-15</td><td>3</td><td>IGHJ6</td><td>9</td><td>CARDADGMDVW</td><td>2</td><td>major</td><td>698</td></tr>
<tr><td>LC-MBL_6</td><td>LC-MBL_6.1_H</td><td>29.32</td><td>IGHV4-34</td><td>95.79</td><td>IGHD3-10</td><td>3</td><td>IGHJ4</td><td>8</td><td>CARRGGVDYW</td><td>V4.6|J4.6|8|1</td><td>minor</td><td>14</td></tr>
</table>

LC-MBL_6	LC-MBL_6.3_H	15.55	IGHV3-72	92.52	IGHD3-22	2	IGHJ3	19	CVRVGDYFDSSGSSLDALDFW	V3-72|J3|19|1	minor	5
LC-MBL_6	LC-MBL_6.8_H	2.33	IGHV4-34	93.33	IGHD1-26	3	IGHJ4	15	CARGEGSGSYYPYFDSW	11	minor	18
LC-MBL_6	LC-MBL_6.9_H	2.19	IGHV4-34	93.33	IGHD3-10	2	IGHJ4	15	CARGDSSGSYYSPFDHW	11	minor	18
LC-MBL_6	LC-MBL_6.13_H	1.46	IGHV4-59	98.95	IGHD2-8	3	IGHJ4	11	CARVRPVSYCDYW	V4|J4|11|2	minor	36
LC-MBL_6	LC-MBL_6.15_H	1.34	IGHV3-74	94.79	IGHD2-8	2	IGHJ5	19	CAREGGDYCTNLLCWNWFDPW	novel subset 8	minor	2
LC-MBL_8	LC-MBL_8.1_H	43.80	IGHV3-15	93.88	IGHD1-26	1	IGHJ4	14	CSTDRPFSGATTFDYW	novel subset 9	minor	2
LC-MBL_8	LC-MBL_8.2_H	30.95	IGHV3-43	97.57	IGHD6-13	1	IGHJ4	14	CAKELGRRSWSEVDSW	novel subset 10	minor	3
LC-MBL_8	LC-MBL_8.3_H	7.02	IGHV4-34	89.82	IGHD6-19	2	IGHJ6	14	CGAVAAVREYYGMDVW	V4|J6|14|10	minor	16
LC-MBL_8	LC-MBL_8.5_H	2.05	IGHV3-74	98.61	IGHD6-25	3	IGHJ6	15	CARDRRIKHYYYGMDVW	V3-30-3|J4.6|15|1	minor	10
LC-MBL_8	LC-MBL_8.6_H	1.58	IGHV3-43	95.49	IGHD6-19	1	IGHJ4	14	CAKELNNRDWSEIDSW	novel subset 10	minor	3
LC-MBL_8	LC-MBL_8.7_H	1.47	IGHV3-15	94.22	IGHD2-21	2	IGHJ1	18	CTVQTLGFCGGVCYSPTNYW	novel subset 11	minor	2
LC-MBL_8	LC-MBL_8.8_H	1.31	IGHV3-30	88.54	IGHD1-26	3	IGHJ4	12	CVRDISGSYSFDYW	V3|J4|12|33	minor	24
LC-MBL_9	LC-MBL_9.2_H	5.02	IGHV3-21	100	IGHD1-26	3	IGHJ3	9	CARDGDAFDIW	novel subset 12	minor	2
LC-MBL_9	LC-MBL_9.4_H	1.83	IGHV3-48	99.65	IGHD3-16	3	IGHJ4	8	CARGGVMGYW	V3-7|J4|8|6	minor	2
LC-MBL_9	LC-MBL_9.7_H	1.11	IGHV3-23	95.83	IGHD4-23	2	IGHJ4	13	CVRRSGANSGLFDSW	novel subset 13	minor	2
LC-MBL_9	LC-MBL_9.8_H	1.05	IGHV3-48	92.98	IGHD5-12	3	IGHJ4	13	CARDAGYSGYVFDSW	novel subset 14	minor	2
LC-MBL_9	LC-MBL_9.10_H	0.94	IGHV3-33	95.49	IGHD4-17	3	IGHJ4	12	CARDNTVTTGLDYW	novel subset 15	minor	3
LC-MBL_10	LC-MBL_10.2_H	6.53	IGHV1-8	97.22	IGHD3-3	3	IGHJ5	17	CARAPRRAGIIIKDWFDPW	novel subset 16	minor	2
LC-MBL_10	LC-MBL_10.4_H	4.99	IGHV3-30	93.03	IGHD2-21	2	IGHJ6	9	CAKDSIPMDVW	V3-30|J6|9|1	minor	2
LC-MBL_10	LC-MBL_10.9_H	1.60	IGHV3-7	97.57					CARDNQGLDQW	V3|J6|9|5	minor	7
LC-MBL_10	LC-MBL_10.10_H	1.45	IGHV3-23	87.85	IGHD6-13	2	IGHJ5	14	CAKGAAPDKVDWFDPW	novel subset 17	minor	2
LC-MBL_10	LC-MBL_10.13_H	1.36	IGHV3-73	93.54	IGHD4-11	2	IGHJ5	9	CARQDSSIDSW	novel subset 18	minor	2
LC-MBL_11	LC-MBL_11.4_H	3.93	IGHV3-7	96.53	IGHD1-1	1	IGHJ4	7	CVNTGFDYW	novel subset 19	minor	2
LC-MBL_11	LC-MBL_11.5_H	3.51	IGHV3-48	93.06	IGHD1-1	3	IGHJ3	10	CVRDYMYAFDIW	novel subset 20	minor	9
LC-MBL_11	LC-MBL_11.7_H	2.54	IGHV4-34	93.68	IGHD4-23	2	IGHJ4	13	CMRGGGNSGYYFDYW	novel subset 21	minor	2
LC-MBL_12	LC-MBL_12.1_H	75.72	IGHV1-8	96.18	IGHD6-13	2	IGHJ4	10	CARGLADNTDYW	V1-8|J4.5|10|1	minor	5
LC-MBL_12	LC-MBL_12.2_H	5.63	IGHV3-48	97.57	IGHD3-16	1	IGHJ4	9	CARGGFDGDYW	novel subset 22	minor	2
LC-MBL_12	LC-MBL_12.4_H	2.59	IGHV3-7	88.89	IGHD1-7	1	IGHJ4	12	CARLLGSGTTFDYW	novel subset 23	minor	3
LC-MBL_12	LC-MBL_12.5_H	1.28	IGHV3-33	100	IGHD3-22	2	IGHJ6	22	CATQYYYDSSGYYWLYYYYGMDVW	V3|J4.6|22|12	minor	9
LC-MBL_12	LC-MBL_12.6_H	1.20	IGHV3-7	95.49	IGHD3-16	2	IGHJ4	11	CARVRGSYSLDFW	novel subset 24	minor	3
LC-MBL_12	LC-MBL_12.8_H	1.06	IGHV3-23	92.36	IGHD3-22	2	IGHJ4	16	CAKEPGDSTGPNHYLDYW	novel subset 25	minor	2
LC-MBL_13	LC-MBL_13.3_H	2.48	IGHV4-34	90.04	IGHD1-26	2	IGHJ3	17	CARRPEHWTEQLGDGFDVW	201	major	95

LC-MBL_13	LC-MBL_13.4_H	2.33	IGHV3-49	89.8	IGHD5-18	3	IGHJ4	7	CAHSYGFYW	novel subset 26	minor	2
LC-MBL_13	LC-MBL_13.7_H	1.88	IGHV4-4	95.49	IGHD3-22	2	IGHJ4	13	CARHADSSGYYSDYW	novel subset 4	minor	4
LC-MBL_13	LC-MBL_13.20_H	1.19	IGHV5-51	99.31	-	-	IGHJ6	8	CARRDYMDVW	V1.5|J6|8|1	minor	4
LC-MBL_13	LC-MBL_13.22_H	1.12	IGHV3-30	95.83	IGHD3-10	2	IGHJ6	19	CARRMGYGSGSYSYYYGMDVW	novel subset 27	minor	2
LC-MBL_13	LC-MBL_13.23_H	1.04	IGHV3-11	93.06	IGHD1-1	3	IGHJ6	10	CARAYWNAMDVW	V3|J6|10|30	minor	32
LC-MBL_13	LC-MBL_13.24_H	1.00	IGHV3-30	90.18	IGHD3-22	2	IGHJ4	11	CAKDSDSWGFDYW	novel subset 28	minor	16
LC-MBL_14	LC-MBL_14.1_H	69.73	IGHV1-2	92.36	IGHD1-26	3	IGHJ4	10	CARDTSGSCVYW	novel subset 29	minor	2
LC-MBL_14	LC-MBL_14.3_H	2.30	IGHV3-21	96.88	IGHD3-16	2	IGHJ6	9	CASDRNGMDVW	2	major	698
LC-MBL_15	LC-MBL_15.1_H	4.85	IGHV3-7	97.22	IGHD1-26	2	IGHJ5	11	CARDGWDNLYDYW	novel subset 30	minor	2
LC-MBL_15	LC-MBL_15.3_H	2.37	IGHV3-7	96.88	IGHD6-19	1	IGHJ4	9	CARGSSGLGYW	novel subset 31	minor	2
LC-MBL_15	LC-MBL_15.5_H	1.67	IGHV3-7	97.57	IGHD6-19	2	IGHJ4	8	CARGVAVADW	novel subset 32	minor	2
LC-MBL_15	LC-MBL_15.7_H	1.55	IGHV3-7	94.44	IGHD2-15	2	IGHJ4	16	CARDVPNCSGGSCYFDYW	novel subset 33	minor	2
LC-MBL_15	LC-MBL_15.9_H	1.17	IGHV3-73	92.52	IGHD3-3	2	IGHJ3	20	CTSENTYYDFWSGYYSGAFDIW	V3|J3.4.6|20|41	minor	10
LC-MBL_15	LC-MBL_15.11_H	0.96	IGHV3-74	98.26	IGHD4-23	3	IGHJ4	10	CARVTMGAFDYW	V3-23|J4|10|2	minor	2
LC-MBL_16	LC-MBL_16.3_H	1.26	IGHV4-34	97.19	IGHD6-6	1	IGHJ4	13	CARGHSSSPYWVDYW	novel subset	minor	2
LC-MBL_16	LC-MBL_16.4_H	1.07	IGHV3-30	89.86	IGHD3-22	2	IGHJ4	11	CAKDSDSWGFDYW	novel subset 28	minor	16
LC-MBL_17	LC-MBL_17.5_H	3.25	IGHV3-23	96.53	IGHD3-22	2	IGHJ4	15	CAKDRYDNSDCYHFDYW	novel subset 34	minor	3
LC-MBL_17	LC-MBL_17.6_H	1.99	IGHV3-7	97.57	IGHD3-16	1	IGHJ4	11	CAREGPARSLDYW	V3|J1.3.4.5|11|48	minor	65
LC-MBL_17	LC-MBL_17.8_H	1.39	IGHV3-72	91.5	IGHD1-26	1	IGHJ4	12	CARSPVGTTPFDYW	novel subset 35	minor	3
LC-MBL_20	LC-MBL_20.1_H	64.79	IGHV3-48	94.68	IGHD3-10	2	IGHJ4	15	CARYGSGSYYRDPFDYW	novel subset 36	minor	4
LC-MBL_20	LC-MBL_20.3_H	11.44	IGHV3-11	93.62	IGHD3-10	2	IGHJ4	15	CARYGSGSYYRDPFDYW	novel subset 36	minor	4
LC-MBL_21	LC-MBL_21.3_H	94.47	IGHV3-30	92.01	IGHD6-19	1	IGHJ4	12	CAKDLTNAWSLDYW	novel subset 38	minor	28
LC-MBL_22	LC-MBL_22.1_H	98.27	IGHV3-74	97.92	IGHD6-19	2	IGHJ4	16	CARGIAVAGSAWYYFDYW	V3-30|J4|16|2	minor	4

Supplementary Table 4. Basic information for all individual CLL cases included in the comparative analysis of intraclonal diversification between MBL and CLL.

Sample ID	Clonotype ID	Group	IG chain	IGHV gene	IGHV germline identity %	IGHD gene	IGHD RF	IGHJ gene	CDR3 length	CDR3 aa sequence	Sequences, No	Sequences, %
CLL_sub2_1_H	CLL_sub2_1.1_H	CLL #2	heavy	IGHV3-21	98.61			IGHJ6	9	CARDQNAMDVW	153331	99.57
CLL_sub2_2_H	CLL_sub2_2.1_H	CLL #2	heavy	IGHV3-21	97.57			IGHJ6	9	CARDANGMDVW	101094	99.49
CLL_sub2_3_H	CLL_sub2_3.1_H	CLL #2	heavy	IGHV3-21	96.18			IGHJ6	9	CVTDRNGMDVW	210663	99.96
CLL_sub2_4_H	CLL_sub2_4.1_H	CLL #2	heavy	IGHV3-21	98.61			IGHJ6	9	CARDQNAMDVW	160472	99.91
CLL_sub2_5_H	CLL_sub2_5.1_H	CLL #2	heavy	IGHV3-21	98.95	IGHD5-12	1	IGHJ6	9	CATDRNGMDVW	170192	98.19
CLL_sub2_6_H	CLL_sub2_6.1_H	CLL #2	heavy	IGHV3-21	97.22	IGHD3-16	2	IGHJ6	9	CASDRNGMDVW	186019	99.95
CLL_sub2_7_H	CLL_sub2_7.1_H	CLL #2	heavy	IGHV3-21	97.92	IGHD2-2	2	IGHJ6	9	CARDANGMDVW	179102	99.87
CLL_sub2_8_H	CLL_sub2_8.1_H	CLL #2	heavy	IGHV3-21	97.92			IGHJ6	9	CARDQNAMDVW	168164	92.08
CLL_sub2_9_H	CLL_sub2_9.1_H	CLL #2	heavy	IGHV3-21	96.18			IGHJ6	9	CARDQNGMDVW	129878	99.85
CLL_sub2_10_H	CLL_sub2_10.1_H	CLL #2	heavy	IGHV3-21	97.89	IGHD1-26	3	IGHJ6	9	CAGDANGMDVW	167048	99.72
CLL_sub2_11_H	CLL_sub2_11.1_H	CLL #2	heavy	IGHV3-21	100	IGHD4-17	2	IGHJ6	9	CARDENVMDVW	124661	99.87
CLL_sub2_12_H	CLL_sub2_12.1_H	CLL #2	heavy	IGHV3-21	98.95			IGHJ6	9	CARDQTDMDVW	155872	99.82
CLL_sub2_13_H	CLL_sub2_13.1_H	CLL #2	heavy	IGHV3-21	99.3			IGHJ6	9	CARDQNVMDVW	124886	99.89
CLL_sub2_14_H	CLL_sub2_14.1_H	CLL #2	heavy	IGHV3-21	98.25	IGHD1-1	1	IGHJ6	9	CARDANGMDVW	134291	99.98
CLL_sub2_15_H	CLL_sub2_15.1_H	CLL #2	heavy	IGHV3-21	97.22	IGHD2-15	2	IGHJ6	9	CARDGNGMDVW	164961	99.92
CLL_sub2_16_H	CLL_sub2_16.1_H	CLL #2	heavy	IGHV3-21	94.79	IGHD1-1	1	IGHJ6	9	CASDRTGMDIW	132712	98.67
CLL_sub2_17_H	CLL_sub2_17.1_H	CLL #2	heavy	IGHV3-21	97.57	IGHD1-14	3	IGHJ6	9	CARDRNGMDVW	146038	97.24
CLL_sub2_18_H	CLL_sub2_18.1_H	CLL #2	heavy	IGHV3-21	99.31	IGHD1-1	1	IGHJ6	9	CARDANGMDVW	155156	99.76
CLL_sub2_19_H	CLL_sub2_19.1_H	CLL #2	heavy	IGHV3-21	97.57	IGHD2-15	3	IGHJ6	9	CARDADGMDVW	151485	99.70
CLL_sub2_2_L	CLL_sub2_2.1_L	CLL #2	light	IGLV3-21	99.64			IGLJ3	12	CQVWDSSSDHPWVF	342960	99.78
CLL_sub2_3_L	CLL_sub2_3.1_L	CLL #2	light	IGLV3-21	98.92			IGLJ3	12	CQVWDSGSDHPWVF	195327	99.75
CLL_sub2_4_L	CLL_sub2_4.1_L	CLL #2	light	IGLV3-21	98.21			IGLJ3	12	CQVWDSGSDHPWVF	204974	99.87
CLL_sub2_5_L	CLL_sub2_5.1_L	CLL #2	light	IGLV3-21	97.13			IGLJ3	12	CQVWDSGSDHPWVF	162982	99.94
CLL_sub2_6_L	CLL_sub2_6.1_L	CLL #2	light	IGLV3-21	98.57			IGLJ3	12	CQVWDSGSDHPWVF	176256	98.91
CLL_sub2_7_L	CLL_sub2_7.1_L	CLL #2	light	IGLV3-21	98.57			IGLJ3	12	CQVWDSSDDHPWVF	177781	96.45

CLL_sub2_8_L	CLL_sub2_8.1_L	CLL #2	light	IGLV3-21	98.57			IGLJ3	12	CQVWDSSSDHPWVF	165762	99.91
CLL_sub2_9_L	CLL_sub2_9.1_L	CLL #2	light	IGLV3-21	98.92			IGLJ3	12	CQVWDSSSDHPWVF	209391	83.61
CLL_sub2_10_L	CLL_sub2_10.1_L	CLL #2	light	IGLV3-21	99.28			IGLJ3	12	CQVWDSGSDQPWVF	210469	96.90
CLL_sub2_11_L	CLL_sub2_11.1_L	CLL #2	light	IGLV3-21	98.21			IGLJ3	12	CQVWDSGSDHPWVF	224119	99.88
CLL_sub2_12_L	CLL_sub2_12.1_L	CLL #2	light	IGLV3-21	98.57			IGLJ3	12	CQVWDSSSDHPWVF	230576	99.72
CLL_sub2_13_L	CLL_sub2_13.1_L	CLL #2	light	IGLV3-21	98.92			IGLJ3	12	CQVWDSGSDHPWVF	186514	99.91
CLL_sub2_14_L	CLL_sub2_14.1_L	CLL #2	light	IGLV3-21	97.85			IGLJ3	12	CQVWDSSSDHPWVF	177622	99.27
CLL_sub2_15_L	CLL_sub2_15.1_L	CLL #2	light	IGLV3-21	98.57			IGLJ3	12	CQVWDSSSDHPWVF	163454	99.79
CLL_sub2_16_L	CLL_sub2_16.1_L	CLL #2	light	IGLV3-21	99.28			IGLJ3	12	CQVWDSSSDHPWVF	234757	99.93
CLL_sub2_17_L	CLL_sub2_17.1_L	CLL #2	light	IGLV3-21	98.21			IGLJ3	12	CQVWDGSSDHPWVF	373847	99.89
CLL_sub2_18_L	CLL_sub2_18.1_L	CLL #2	light	IGLV3-21	99.28			IGLJ3	12	CQVWDSGSDHPWVF	507690	99.95
CLL_sub2_19_L	CLL_sub2_19.1_L	CLL #2	light	IGLV3-21	99.64			IGLJ3	12	CQVWDSSSDHPWVF	299672	99.82
CLL_sub2_20_L	CLL_sub2_20.1_L	CLL #2	light	IGLV3-21	97.13			IGLJ3	12	CQVWDSGSDQPWVF	284539	99.53
CLL_sub2_21_L	CLL_sub2_21.1_L	CLL #2	light	IGLV3-21	99.28			IGLJ3	12	CQVWDSGSDHPWVF	306372	99.92
CLL_sub2_22_L	CLL_sub2_22.1_L	CLL #2	light	IGLV3-21	98.92			IGLJ3	12	CQMWDSGSDHPWVF	212928	99.44
CLL_sub4_1_H	CLL_sub4_1.1_H	CLL #4	heavy	IGHV4-34	93.33	IGHD4-17	2	IGHJ6	20	CARGYGDSADTKRYYYFGLDVW	112811	52.27
CLL_sub4_2_H	CLL_sub4_2.1_H	CLL #4	heavy	IGHV4-34	96.14	IGHD4-17	2	IGHJ6	20	CARGYGDSPDIKRYYYYGLDVW	225141	51.50
CLL_sub4_3_H	CLL_sub4_3.1_H	CLL #4	heavy	IGHV4-34	95.44	IGHD5-18	1	IGHJ6	20	CARGYPDTPVVRRYYYYGMDVW	299932	98.07
CLL_sub4_4_H	CLL_sub4_4.1_H	CLL #4	heavy	IGHV4-34	95.44	IGHD5-18	1	IGHJ6	20	CARGYPDTPVVRRYYYYGMDVW	225623	97.02
CLL_sub4_5_H	CLL_sub4_5.1_H	CLL #4	heavy	IGHV4-34	95.44	IGHD5-18	1	IGHJ6	20	CARGYPDTPVVRRYYYYGMDVW	431514	93.39
CLL_sub4_6_H	CLL_sub4_6.1_H	CLL #4	heavy	IGHV4-34	92.63	IGHD5-18	1	IGHJ6	20	CARGYADTPTFRRYYYYGMDVW	257937	82.11
CLL_sub4_7_H	CLL_sub4_7.1_H	CLL #4	heavy	IGHV4-34	92.63	IGHD5-18	1	IGHJ6	20	CARGYADTPTFRRYYYYGMDVW	96828	53.00
CLL_sub4_8_H	CLL_sub4_8.1_H	CLL #4	heavy	IGHV4-34	92.63	IGHD5-18	1	IGHJ6	20	CARGYADTPTFRRYYYYGMDVW	366766	37.12
CLL_sub4_9_H	CLL_sub4_9.1_H	CLL #4	heavy	IGHV4-34	91.58	IGHD2-15	3	IGHJ6	20	CARGYADSDVIRRYYYYGMDVW	231156	95.68
CLL_sub4_10_H	CLL_sub4_10.1_H	CLL #4	heavy	IGHV4-34	91.58	IGHD2-15	3	IGHJ6	20	CARGYADSDVIRRYYYYGMDVW	663276	99.62
CLL_sub4_11_H	CLL_sub4_11.1_H	CLL #4	heavy	IGHV4-34	91.58	IGHD2-15	3	IGHJ6	20	CARGYADSDVIRRYYYYGMDVW	758812	99.31
CLL_sub4_12_H	CLL_sub4_12.1_H	CLL #4	heavy	IGHV4-34	91.58	IGHD2-15	3	IGHJ6	20	CARGYADSDVIRRYYYYGMDVW	544720	95.91
CLL_sub4_13_H	CLL_sub4_13.1_H	CLL #4	heavy	IGHV4-34	93.33	IGHD4-17	3	IGHJ6	20	CARGYGTSATTKRYYYYGMDVW	317486	99.42
CLL_sub4_14_H	CLL_sub4_14.1_H	CLL #4	heavy	IGHV4-34	93.33	IGHD4-17	3	IGHJ6	20	CARGYGTSATTKRYYYYGMDVW	475068	99.25
CLL_sub4_15_H	CLL_sub4_15.1_H	CLL #4	heavy	IGHV4-34	93.33	IGHD4-17	3	IGHJ6	20	CARGYGTSATTKRYYYYGMDVW	209149	99.49
CLL_sub4_16_H	CLL_sub4_16.1_H	CLL #4	heavy	IGHV4-34	93.33	IGHD4-17	3	IGHJ6	20	CARGYGTSATTKRYYYYGMDVW	377868	99.08
CLL_sub4_17_H	CLL_sub4_17.1_H	CLL #4	heavy	IGHV4-34	93.33	IGHD4-17	3	IGHJ6	20	CARGYGTSATTKRYYYYGMDVW	214103	98.85

CLL_sub4_18_H	CLL_sub4_18.1_H	CLL #4	heavy	IGHV4-34	89.44	IGHD3-10	2	IGHJ6	20	CARGYGTSDDTRRYYFYGMDVW	462708	99.87
CLL_sub4_3_K	CLL_sub4_3.1_K	CLL #4	light	IGKV2-30	96.6			IGKJ2	10	CMQGTHGPPYTF	96157	98.48
CLL_sub4_4_K	CLL_sub4_4.1_K	CLL #4	light	IGKV2-30	98.64			IGKJ2	10	CMQGTHWPPYTF	122444	85.25
CLL_sub4_5_K	CLL_sub4_5.1_K	CLL #4	light	IGKV2-30	97.28			IGKJ2	9	CMQGTHWPYTF	283712	93.82
CLL_sub4_6_K	CLL_sub4_6.1_K	CLL #4	light	IGKV2-30	95.92			IGKJ2	9	CMQGTHWPYTF	11006	94.90
CLL_sub4_7_K	CLL_sub4_7.1_K	CLL #4	light	IGKV2-30	97.28			IGKJ2	9	CMQGTHWPYTF	11407	83.83
CLL_sub4_8_K	CLL_sub4_8.1_K	CLL #4	light	IGKV2-30	97.28			IGKJ2	9	CMQGTYWPYNF	209197	99.15
CLL_sub4_9_K	CLL_sub4_9.1_K	CLL #4	light	IGKV2-30	95.58			IGKJ1	9	CMQGTHWPPTF	227018	94.41
CLL_sub4_10_K	CLL_sub4_10.1_K	CLL #4	light	IGKV2-30	99.66			IGKJ2	9	CMQGTHWPYTF	247596	73.81
CLL_nonsub_1_H	CLL_nonsub_1.1_H	CLL non-subset	heavy	IGHV4-34	92.63	IGHD6-13	2	IGHJ4	15	CARGAAAGTWAGGFDFW	235194	99.68
CLL_nonsub_2_H	CLL_nonsub_2.1_H	CLL non-subset	heavy	IGHV4-34	92.63	IGHD6-13	2	IGHJ4	15	CARGAAAGTWAGGFDFW	393412	99.54
CLL_nonsub_3_H	CLL_nonsub_3.1_H	CLL non-subset	heavy	IGHV4-34	92.83	IGHD1-26	3	IGHJ6	19	CARGPIQKSGRNFYYYYMDVW	195264	97.50
CLL_nonsub_4_H	CLL_nonsub_4.1_H	CLL non-subset	heavy	IGHV4-34	92.83	IGHD1-26	3	IGHJ6	19	CARGPIQKSGRNFYYYYMDVW	116351	96.74
CLL_nonsub_5_H	CLL_nonsub_5.1_H	CLL non-subset	heavy	IGHV4-34	95.44	IGHD7-27	1	IGHJ4	13	CARGETGARVPLVYW	145988	97.94
CLL_nonsub_6_H	CLL_nonsub_6.1_H	CLL non-subset	heavy	IGHV4-34	95.44	IGHD7-27	1	IGHJ4	13	CARGETGARVPLVYW	132783	97.32
CLL_nonsub_7_H	CLL_nonsub_7.1_H	CLL non-subset	heavy	IGHV4-34	95.44	IGHD4-11	2	IGHJ4	14	CARGDYSDYVSRPDYW	89717	99.14
CLL_nonsub_8_H	CLL_nonsub_8.1_H	CLL non-subset	heavy	IGHV4-34	95.44	IGHD4-11	2	IGHJ4	14	CARGDYSDYVSRPDYW	112143	99.93
CLL_nonsub_9_H	CLL_nonsub_9.1_H	CLL non-subset	heavy	IGHV4-34	97.19	IGHD2-2	3	IGHJ4	11	CARGNLLRAFDCW	93791	66.37
CLL_nonsub_10_H	CLL_nonsub_10.1_H	CLL non-subset	heavy	IGHV4-34	97.19	IGHD2-2	3	IGHJ4	11	CARGNLLRAFDCW	215708	83.01
CLL_nonsub_11_H	CLL_nonsub_11.1_H	CLL non-subset	heavy	IGHV4-34	94.04	IGHD6-6	3	IGHJ4	11	CATRGQVGAFRYW	222185	99.30
CLL_nonsub_12_H	CLL_nonsub_12.1_H	CLL non-subset	heavy	IGHV4-34	94.04	IGHD6-6	3	IGHJ4	11	CATRGQVGAFRYW	161877	99.45
CLL_nonsub_13_H	CLL_nonsub_13.1_H	CLL non-subset	heavy	IGHV7-4-1	96.88	IGHD3-10	3	IGHJ5	20	CARDVGSMVRGVIINPRWFDPW	263463	99.47
CLL_nonsub_14_H	CLL_nonsub_14.1_H	CLL non-subset	heavy	IGHV3-53	97.54	IGHD6-19	1	IGHJ4	15	CARDPVSSGWYGAFDYW	262270	97.47
CLL_nonsub_15_H	CLL_nonsub_15.1_H	CLL non-subset	heavy	IGHV4-59	100	IGHD3-3	2	IGHJ6	24	CARVPPAYDFWSGYYGVAYYYYMDVW	70613	98.81
CLL_nonsub_16_H	CLL_nonsub_16.1_H	CLL non-subset	heavy	IGHV3-11	97.57	IGHD6-19	1	IGHJ4	14	CARDFGSSGSGTYACW	212002	98.19
CLL_nonsub_17_H	CLL_nonsub_17.1_H	CLL non-subset	heavy	IGHV1-2	100	IGHD2-8	3	IGHJ2	25	CARSRDIVLMVYAIQPNKNGVRYFDLW	71835	98.85
CLL_nonsub_18_H	CLL_nonsub_18.1_H	CLL non-subset	heavy	IGHV4-61	92.71	IGHD5-24	3	IGHJ5	16	CARSHDINYYQAAGFGPW	92393	99.27
CLL_nonsub_19_H	CLL_nonsub_19.1_H	CLL non-subset	heavy	IGHV3-53	87.02	IGHD1-7	3	IGHJ4	16	CARGRYERNTYYFSYEYW	51544	93.67
CLL_nonsub_20_H	CLL_nonsub_20.1_H	CLL non-subset	heavy	IGHV3-20	95.49	IGHD2-2	2	IGHJ4	20	CARSEWRYCSSTDCHRVNFDYW	79983	99.96
CLL_nonsub_21_H	CLL_nonsub_21.1_H	CLL non-subset	heavy	IGHV3-7	90.62	IGHD3-16	3	IGHJ4	13	CARGGDTAYRPSDYW	63082	85.84
CLL_nonsub_22_H	CLL_nonsub_22.1_H	CLL non-subset	heavy	IGHV4-34	98.95	IGHD2-15	2	IGHJ6	26	CARTPTDYCSGGSCYSVGEVDYYGMDVW	92221	99.82
CLL_nonsub_23_H	CLL_nonsub_23.1_H	CLL non-subset	heavy	IGHV3-49	94.56	IGHD3-3	2	IGHJ4	16	CTRDEGSGYFKFSGFDYW	60331	98.87

CLL_nonsub_24_H	CLL_nonsub_24.1_H	CLL non-subset	heavy	IGHV1-18	99.31	IGHD3-16	1	IGHJ4	10	CARDSGGSVDYW	64931	99.96
CLL_nonsub_25_H	CLL_nonsub_25.1_H	CLL non-subset	heavy	IGHV3-7	90.97	IGHD4-11	2	IGHJ4	7	CTRGHYEIW	88515	99.44
CLL_nonsub_26_H	CLL_nonsub_26.1_H	CLL non-subset	heavy	IGHV3-11	98.61	IGHD6-6	1	IGHJ6	20	CARDGQYSSSVGYYYYYGTDVW	20926	99.75
CLL_nonsub_27_H	CLL_nonsub_27.1_H	CLL non-subset	heavy	IGHV1-69	97.57	IGHD6-13	1	IGHJ4	15	CARSMDSSSWGGAFDYW	42921	80.60
CLL_nonsub_28_H	CLL_nonsub_28.1_H	CLL non-subset	heavy	IGHV4-34	89.82	IGHD3-3	2	IGHJ4	22	CARHNKGYFDLWSGSRGGGNFDYW	220743	97.44
CLL_nonsub_29_H	CLL_nonsub_29.1_H	CLL non-subset	heavy	IGHV3-48	92.01	IGHD5-18	1	IGHJ4	17	CANPEGDDTSMGRPLFDYW	268395	95.41
CLL_nonsub_30_H	CLL_nonsub_30.1_H	CLL non-subset	heavy	IGHV1-3	92.36	IGHD2-15	2	IGHJ5	20	CATAPPRGCSGGICYSAWFDTW	529349	99.84
CLL_nonsub_31_H	CLL_nonsub_31.1_H	CLL non-subset	heavy	IGHV3-74	94.79	IGHD1-26	1	IGHJ4	16	CARAPIVEATTGGGFDYW	544653	99.70
CLL_nonsub_32_H	CLL_nonsub_32.1_H	CLL non-subset	heavy	IGHV2-5	95.88	IGHD3-3	2	IGHJ5	21	CAHMRREYDFWSGSHIPNWFDPW	21735	97.66
CLL_nonsub_33_H	CLL_nonsub_33.1_H	CLL non-subset	heavy	IGHV3-74	97.22	IGHD6-19	1	IGHJ4	11	CARGFSGWLQDYW	412082	99.77
CLL_nonsub_34_H	CLL_nonsub_34.1_H	CLL non-subset	heavy	IGHV3-23	90.62	IGHD6-19	1	IGHJ4	17	CAKGSAGAGSTTGWPLDHW	261984	92.51
CLL_nonsub_35_H	CLL_nonsub_35.1_H	CLL non-subset	heavy	IGHV3-33	90.97			IGHJ6	9	CARDSYGMDVW	17531	98.57
CLL_nonsub_36_H	CLL_nonsub_36.1_H	CLL non-subset	heavy	IGHV3-33	93.75	IGHD1-26	3	IGHJ6	18	CARGVSGGRSRYYYYAIDVW	219674	99.18
CLL_nonsub_37_H	CLL_nonsub_37.1_H	CLL non-subset	heavy	IGHV3-7	96.18	IGHD2-2	3	IGHJ5	14	CATCVVVPAAMNFDPW	378995	94.80
CLL_nonsub_38_H	CLL_nonsub_38.1_H	CLL non-subset	heavy	IGHV4-59	92.28	IGHD3-3	1	IGHJ2	13	CARDGVGWDLPFDLW	170312	92.38
CLL_nonsub_39_H	CLL_nonsub_39.1_H	CLL non-subset	heavy	IGHV3-23	93.06	IGHD3-16	3	IGHJ3	17	CAKVRRFSGAASGDAFDIW	22858	25.20
CLL_nonsub_40_H	CLL_nonsub_40.1_H	CLL non-subset	heavy	IGHV3-48	97.92	IGHD5-12	1	IGHJ4	10	CARGGTVETNNW	693644	99.84
CLL_nonsub_41_H	CLL_nonsub_41.1_H	CLL non-subset	heavy	IGHV3-23	87.5	IGHD1-1	2	IGHJ4	18	CAKDSREQPVENLELLIDSW	234731	98.91
CLL_nonsub_1_L	CLL_nonsub_1.1_L	CLL non-subset	light	IGLV2-8	96.18			IGLJ1	10	CSSYAGSNRGVF	336835	99.70
CLL_nonsub_2_K	CLL_nonsub_2.1_K	CLL non-subset	light	IGKV3-11	96.06			IGKJ4	11	CQQRSNWPPALTF	150745	99.88
CLL_nonsub_3_K	CLL_nonsub_3.1_K	CLL non-subset	light	IGKV3-15	96.77			IGKJ1	9	CQQYDNWPRTF	172004	99.26
CLL_nonsub_4_K	CLL_nonsub_4.1_K	CLL non-subset	light	IGKV3-20	97.87			IGKJ4	9	CQQSGSSPLTF	188670	99.74
CLL_nonsub_5_K	CLL_nonsub_5.1_K	CLL non-subset	light	IGKV4-1	97.64			IGKJ2	10	CQQYYTTPPCTF	394545	92.82
CLL_nonsub_6_L	CLL_nonsub_6.1_L	CLL non-subset	light	IGLV2-14	95.49			IGLJ3	10	CSSYTSSNTRVF	253644	99.70

Supplementary Table 5. Mean values for all graph network metrics in the 5 distinct sample groups included in the analysis of intraclonal diversification.

IG chain	Group	Types of average	Relative reads convergence	End nodes density	Maximal pathway length	Maximal mutational length	Average degree	Average distance
heavy	oligoclonal MBL	mean	0.166	0.447	2.42	3.89	2.02	1.79
		median	0.019	0.444	2	3	2	2
		min	0.004	0.200	2	2	1.33	1
		max	1.048	0.722	5	8	2.48	3
	monoclonal MBL	mean	0.108	0.325	2.67	4.17	2.36	1.50
		median	0.014	0.317	2	2.5	2.18	1.5
		min	0.002	0.182	2	2	2	1
		max	0.490	0.500	6	16	4.15	2
	CLL subset #2	mean	0.020	0.248	3.45	6.45	2.55	2.09
		median	0.018	0.250	4	4	2.35	2
		min	0.002	0.098	2	2	2	1
		max	0.061	0.400	5	31	3.62	3
	CLL subset #4	mean	0.006	0.340	2.28	3.56	2.68	2.06
		median	0.005	0.370	2	2.5	2.62	2
		min	0.002	0.167	2	2	2.14	2
		max	0.025	0.421	4	8	3.41	3
	CLL non-subset	mean	0.034	0.344	2.5	4.68	2.47	1.85
		median	0.006	0.333	2	3	2.49	2
		min	0.002	0.111	2	2	1.86	1
		max	0.516	0.615	9	24	4.67	4
light	monoclonal MBL	mean	0.645	0.362	2.44	6.55	2.06	1.39
		median	0.012	0.333	2	5	2.00	1
		min	0.001	0.182	2	2	1.33	1
		max	3.556	0.667	6	20	3.00	2

	**oligoclonal MBL**	mean	0.534	0.356	3.33	5.53	2.04	1.53
		median	0.134	0.400	2	3	2	1
		min	0.012	0.115	2	2	1.5	1
		max	3.822	0.667	8	20	3.37	3
	**CLL subset #2**	mean	0.016	0.237	3.2	8.73	2.86	1.67
		median	0.007	0.200	3	3	2.82	2
		min	0.001	0.061	2	2	2	1
		max	0.082	0.400	7	27	4.44	3
	**CLL subset #4**	mean	0.071	0.280	3	3.4	2.51	1.8
		median	0.004	0.333	2	3	2.46	2
		min	0.003	0.049	2	2	2	1
		max	0.333	0.345	7	7	3.12	2
	**CLL non-subset**	mean	0.006	0.335	2	2	2.46	1.67
		median	0.004	0.333	2	2	2.42	2
		min	0.003	0.333	2	2	2.39	1
		max	0.016	0.342	2	2	2.64	2

Supplementary Table 6. List of the 51 genes included in the GMS Lymphoid gene panel that were also characterized as genetic drivers in large genomic studies in CLL.

ARID1A	*CREBBP*	*IKZF3*	*MYD88*	*SAMHD1*	*ZNF292*
ARID5B	*DDX3X*	*INO80*	*NFKBIE*	*SETD2*	
ASXL1	*DIS3*	*IRF4*	*NOTCH1*	*SF3B1*	
ATM	*EGR2*	*ITPKB*	*NRAS*	*SP140*	
BCOR	*EWSR1*	*KLHL6*	*NSD1*	*SPEN*	
BIRC3	*FAM50A*	*KMT2D*	*PIM1*	*TP53*	
BRAF	*FBXW7*	*KRAS*	*POT1*	*TRAF2*	
CARD11	*GNB1*	*MAP2K1*	*PTPN11*	*TRAF3*	
CCND2	*HIST1H1E*	*MED12*	*RPS15*	*XPO1*	
CHD2	*IKBKB*	*MGA*	*RUFY1*	*ZMYM3*	

Supplementary Table 7. Exonic non-synonymous variants identified in CLL-type MBL and low-count CLL-type MBL.

Sample	Entity	Chr	Start	End	Ref	Var	Gene	Exon	Mutation type	Ref reads	Hotspot position	Var reads	VAF %	cDNA	Protein	HGVS Transcript	HGVS protein
HC-MBL_1	CLL-type MBL	17	7577120	7577120	C	T	*TP53*	8	missense	403	yes	85	21	c.818G>A	p.Arg273His	NM_000546.6	NP_000537.3
HC-MBL_1	CLL-type MBL	17	7578190	7578190	T	C	*TP53*	6	missense	483	yes	11	2.3	c.659A>G	p.Tyr220Cys	NM_000546.6	NP_000537.3
HC-MBL_3	CLL-type MBL	17	7577539	7577539	G	A	*TP53*	7	missense	900	yes	522	58	c.742C>T	p.Arg248Trp	NM_000546.6	NP_000537.3
HC-MBL_5	CLL-type MBL	2	1.98E+08	1.98E+08	G	C	*SF3B1*	16	missense	395	Yes	10	2.5	c.2323C>G	p.Arg775Gly	NM_012433.4	NP_036565.2
HC-MBL_5	CLL-type MBL	3	47147534	47147534	G	A	*SETD2*	6	nonsense	333		12	3.6	c.4792C>T	p.Arg1598Ter	NM_014159.7	NP_054878.5
HC-MBL_5	CLL-type MBL	X	1.54E+08	1.54E+08	A	G	*FAM50A*	12	missense	496		185	3.7	c.950A>G	p.His317Arg	NM_004699.4	NP_004690.1
HC-MBL_8	CLL-type MBL	10	64573335	64573335	C	T	*EGR2*	2	missense	972	yes	467	48.1	c.1063G>A	p.Asp355Asn	NM_000399.5	NP_000390.2
HC-MBL_11	CLL-type MBL	9	1.39E+08	1.39E+08	CAG	C	*NOTCH1*	34	frameshift del	1118	yes	497	44.5	c.7541_7542del	p.Pro2514ArgfsTer4	NM_017617.5	NP_060087.3
HC-MBL_11	CLL-type MBL	10	64573248	64573248	G	T	*EGR2*	2	missense	1024	yes	462	45.1	c.1150C>A	p.His384Asn	NM_000399.5	NP_000390.2
HC-MBL_11	CLL-type MBL	17	7578370	7578370	C	A	*TP53*	5	splicing	700	yes	641	91.6	c.559+1G>T		NM_000546.6	
LC-MBL_6	Low-count CLL-type MBL	6	26156820	26156820	G	C	*HIST1H1E*	1	missense	1102		34	3.1	c.202G>C	p.Ala68Pro	NM_005321.3	NP_005312.1

Chr: Chromosome

Ref: Reference

Var: Variant

frameshift del: frameshift deletion

Supplementary Table 8. Lists of genes exhibiting significant differential regulation among Low-count CLL-type MBL, CLL-type MBL and CLL and the relevant signaling pathways and cellular processes.

	Type of regulation	KEGG Term	Overlap	P-value	Adjusted P-value	Genes
**CLL-type MBL versus Low-count CLL-type MBL**	upregulation	MAPK signaling pathway	75|294	3.12E-06	5.01E-04	*FLT1;FLT4;FGF2;DUSP16;ELK4;RPS6KA4;RPS6KA3;AKT2;KDR;RAC1;HRAS;MAP4K2;PDGFRA;DUSP2;PRKCB;TRAF2;IRAK4;MAPK8IP3;MAPK8IP1;PPM1A;CACNB3;PPM1B;CACNB4;RASA1;KIT;MAPKAPK2;MAPKAPK5;MAPT;RAF1;TP53;MET;SOS2;ATF4;CACNA1B;CACNA1A;NLK;EFNA5;RASGRP2;EFNA4;EGFR;RELB;CACNA1I;MAPK9;PPP3R1;RRAS;ERBB3;ERBB4;ERBB2;GNA12;MKNK2;FLNC;MAP2K7;MAP4K3;MAP2K5;NTRK2;JUND;MAP3K1;TGFB1;TGFB3;BRAF;NFATC1;IGF1;GNG12;NFKB1;NFKB2;MAPK10;MAPK11;FGF18;NF1;TAB2;KRAS;FGFR4;FGFR3;FGFR2;MAP3K12*
**CLL-type MBL versus Low-count CLL-type MBL**	upregulation	PI3K-Akt signaling pathway	78|354	4.20E-04	0.01355752	*CRTC2;CDKN1B;FLT1;FLT4;PTEN;BRCA1;PIK3CB;FGF2;RPTOR;COMP;STK11;AKT2;KDR;RAC1;HRAS;YWHAG;YWHAH;PDGFRA;HSP90AA1;ITGA3;PDPK1;ITGA2;ITGA1;TSC2;PPP2R5A;COL2A1;CCNE1;COL4A1;KIT;COL4A6;COL6A6;COL6A5;RAF1;TP53;SGK2;MET;SOS2;ATF4;PHLPP2;LAMA5;TNXB;LAMA2;LAMA1;LAMA3;EFNA5;THBS2;EFNA4;EGFR;THBS3;VTN;RELN;ERBB3;ERBB4;CD19;CHAD;ERBB2;PCK2;IFNA21;NTRK2;FN1;PPP2R3B;IGF1;GNG12;NFKB1;MTOR;COL1A1;PPP2R2C;GNB2;CDK2;FGF18;PPP2R2D;BCL2;KRAS;COL9A2;PKN1;FGFR4;FGFR3;FGFR2*
**CLL-type MBL versus Low-count CLL-type MBL**	upregulation	Notch signaling pathway	18|59	0.002325	0.03736915	*JAG2;TLE3;NUMBL;TLE1;NOTCH1;MAML2;DTX3L;CTBP1;DTX2;DLL3;LFNG;NCOR2;DLL4;KAT2B;KAT2A;ATXN1L;DVL1;DVL3*
**CLL-type MBL versus Low-count CLL-type MBL**	upregulation	FoxO signaling pathway	31|131	0.007456	0.07996382	*CDKN1B;PTEN;IRS2;PIK3CB;NLK;EGFR;MAPK9;STK11;AKT2;HOMER3;HRAS;S1PR4;PCK2;SMAD4;TGFB1;HOMER2;TGFB3;PDPK1;BRAF;CSNK1E;IGF1;SIRT1;KLF2;MAPK10;MAPK11;CCNG2;CDK2;KRAS;RAF1;SGK2;SOS2*
**CLL-type MBL versus Low-count CLL-type MBL**	upregulation	p53 signaling pathway	19|73	0.011846	0.10526355	*STEAP3;CDKN2A;PTEN;TSC2;TNFRSF10B;IGF1;BBC3;PIDD1;SESN3;CCNE1;CCNG2;ADGRB1;CDK2;BCL2;CDK1;BAX;GTSE1;TP53;TP73*
**CLL-type MBL versus Low-count CLL-type MBL**	upregulation	Wnt signaling pathway	36|166	0.016971	0.12566938	*CAMK2B;WNT2B;CTBP1;CAMK2A;WNT8A;WNT8B;NLK;NKD1;LRP6;WNT6;MAPK9;FRAT1;PPP3R1;FRAT2;ZNRF3;DVL1;DVL3;RAC1;TLE3;WNT10B;SMAD4;TLE1;PRKCB;CSNK2A2;FZD6;FZD8;NFATC2;NFATC1;CSNK1E;MAPK10;PLCB4;CTNNB1;TP53;PLCB2;LGR5;LGR4*
**CLL-type MBL versus Low-count CLL-type MBL**	downregulation	Antigen processing and presentation	28|78	1.99E-08	1.52E-06	*KIR2DL1;IFI30;TNF;KIR2DL3;CTSS;HLA-DMA;B2M;HLA-DOB;HLA-DPA1;PDIA3;CD74;HSPA8;HLA-DRB5;HSPA5;KLRC3;NFYB;HSPA6;KIR2DS4;HLA-G;HLA-E;TAPBP;CD8B;PSME3;PSME1;PSME2;HLA-DRA;KLRD1;HLA-DQB1*
**CLL-type MBL versus Low-count CLL-type MBL**	downregulation	Oxidative phosphorylation	35|133	2.25E-06	7.63E-05	*NDUFB10;UQCRB;NDUFA12;NDUFB5;COX17;NDUFB2;COX7A2;UQCR10;COX11;UQCRFS1;CYC1;NDUFV3;ATP6V1E1;COX10;ATP6V1F;NDUFA8;ATP6V1G1;ATP6V0B;ATP6V0E1;NDUFA6;ATP6AP1;NDUFA5;NDUFA4;NDUFA2;NDUFA1;SDHD;SDHB;COX7A2L;NDUFS5;NDUFS4;UQCRC1;NDUFS2;NDUFS1;UQCRC2;ATP6V0C*
**CLL-type MBL versus Low-count CLL-type MBL**	downregulation	RNA degradation	22|79	6.55E-05	9.99E-04	*HSPA9;ZCCHC7;PARN;LSM1;WDR61;ENO1;LSM3;LSM2;CNOT4;EXOSC5;LSM7;EXOSC10;CNOT7;LSM6;EXOSC9;XRN2;PABPC3;EXOSC3;CNOT8;EXOSC1;DCP1B;MPHOSPH6*
**CLL-type MBL versus Low-count CLL-type MBL**	downregulation	Spliceosome	31|150	9.76E-04	0.008501	*SF3B4;U2AF1L4;SNRPD1;PCBP1;MAGOH;SRSF10;CTNNBL1;HSPA8;PRPF38B;BUD31;HSPA6;THOC3;PRPF40A;THOC2;CWC15;WBP11;LSM3;U2SURP;LSM2;PRPF4;LSM7;HNRNPK;LSM6;SNW1;DDX39B;PPIE;SNRNP27;HNRNPC;SLU7;SNRPA;RBM22*

**CLL-type MBL versus Low-count CLL-type MBL**	downregulation	RNA transport	36|186	0.001405	0.01127427	*POP5;POP7;NXT1;SUMO4;PHAX;SUMO3;TGS1;MAGOH;SUMO2;EIF4EBP1;SAP18;EIF4EBP3;RAE1;EIF2B3;SEC13;EIF2B2;UBE2I;THOC3;THOC2;THOC7;SRRM1;EIF1;NUP93;DDX19B;DDX39B;EIF3I;RPP21;PABPC3;STRAP;EIF3H;RNPS1;EIF3F;EIF4E2;EIF3D;EIF4G2;NUP37*
**CLL-type MBL versus Low-count CLL-type MBL**	downregulation	NF-kappa B signaling pathway	21|104	0.007834	0.04192028	*UBE2I;CXCL8;CSNK2A1;BCL2A1;LY96;TNFAIP3;TNF;RELA;ICAM1;ZAP70;CD40LG;LCK;TRAF6;BTK;BLNK;LTA;TNFSF11;CD14;CARD14;LAT;MYD88*
**CLL versus CLL-type MBL**	upregulation	Notch signaling pathway	18|59	0.014405	0.57078976	*NOTCH2;TLE2;HDAC2;NUMBL;MAML1;CTBP1;HDAC1;NOTCH4;PSEN2;DTX1;RFNG;LFNG;APH1A;APH1B;MFNG;NUMB;DVL2;EP300*
**CLL versus CLL-type MBL**	downregulation	PI3K-Akt signaling pathway	38|354	3.65E-04	0.02636635	*PHLPP2;PRKAA1;LAMA2;LAMA1;LAMA3;LPAR1;LAMC2;EFNA5;EGFR;COMP;RELN;ERBB3;ERBB4;KDR;RAC1;IL6R;EIF4E;EIF4B;PDGFRA;NTRK2;HSP90AA1;ANGPT2;VWF;ANGPT1;PDPK1;GNG12;NFKB1;COL2A1;COL4A1;PPP2R2B;KIT;MDM2;COL6A5;TP53;MET;SOS2;FGFR2;CREB5*
**CLL versus CLL-type MBL**	downregulation	Calcium signaling pathway	27|240	1.23E-03	0.06461794	*RET;RYR1;RYR2;CAMK2A;CACNA1B;EGFR;MYLK3;CACNA1I;PPP3R1;ERBB3;ERBB4;KDR;BDKRB1;CACNA1S;NOS1;PDGFRA;NTRK2;PRKCB;ATP2B3;PHKA1;SLC8A3;P2RX7;PLCB4;ASPH;PLCB1;MET;FGFR2*
**CLL versus CLL-type MBL**	downregulation	MAPK signaling pathway	30|294	3.12E-03	0.07642698	*CACNA1B;EFNA5;EGFR;ELK4;RPS6KA3;CACNA1I;PPP3R1;ERBB3;ERBB4;KDR;RAC1;CACNA1S;PDGFRA;NTRK2;ANGPT2;ANGPT1;PRKCB;RRAS2;GNG12;NFKB1;PPM1B;RASA1;TRAF6;KIT;MAPT;TP53;MET;SOS2;FGFR2;MAP3K12*
**CLL versus Low-count CLL-type MBL**	upregulation	Notch signaling pathway	21|59	0.001537	0.24202958	*TLE3;TLE2;HDAC2;NUMBL;MAML1;CTBP1;NOTCH4;PSEN2;DTX1;DTX2;RFNG;LFNG;NCOR2;APH1A;KAT2A;ATXN1L;DVL1;NUMB;DVL2;EP300;DVL3*
**CLL versus Low-count CLL-type MBL**	upregulation	FoxO signaling pathway	37|131	0.004597	0.25277233	*IRS1;ARAF;AGAP2;PIK3R3;PIK3CD;IRS2;FOXO4;PIK3CB;NLK;IKBKB;MAPK9;STK11;CCNB1;AKT2;AKT1;EP300;HOMER3;HRAS;S1PR4;RAG1;PCK2;PRKAB2;CDKN2B;TGFB1;HOMER2;TGFB3;PLK1;CSNK1E;MAPK14;SIRT1;TGFBR1;MAPK12;MAPK11;CDK2;SGK3;RAF1;SGK2*
**CLL versus Low-count CLL-type MBL**	upregulation	VEGF signaling pathway	19|59	0.008722	0.30120478	*SPHK2;SRC;PLA2G4B;PXN;PIK3CD;PIK3R3;PIK3CB;MAPK14;MAPK12;PPP3CA;MAPK11;MAPKAPK3;AKT2;PLCG2;AKT1;RAC3;PLCG1;RAF1;HRAS*
**CLL versus Low-count CLL-type MBL**	upregulation	Wnt signaling pathway	40|166	0.046134	0.47587944	*INVS;CAMK2B;GSK3B;WNT2B;CTBP1;PRICKLE2;PRICKLE3;WNT8B;PRICKLE1;NLK;NKD1;PPP3CA;MAPK9;CCND3;FRAT1;PORCN;FRAT2;ZNRF3;DVL1;DVL2;EP300;DVL3;RAC3;TLE3;WNT10B;TLE2;WNT10A;FZD2;FZD5;FZD7;CSNK2A2;FZD6;CSNK1E;WNT9A;SENP2;DKK4;APC;LGR6;LGR4;PPARD*
**CLL versus Low-count CLL-type MBL**	downregulation	Antigen processing and presentation	18|78	4.36E-08	1.21E-05	*PDIA3;CD74;HSP90AA1;HSPA5;KLRC3;NFYB;KLRC4;KIR2DL1;KIR3DL2;CTSS;HLA-E;HLA-DMA;CD8B;HLA-DPB1;KLRD1;B2M;HLA-DOB;HLA-DQB1*
**CLL versus Low-count CLL-type MBL**	downregulation	Oxidative phosphorylation	18|133	1.20E-04	0.00371393	*NDUFA9;NDUFA13;ATP6V1G1;NDUFB8;NDUFA7;NDUFA5;SDHC;SDHD;COX6A1;SDHB;ATP4A;COX7A2L;PPA1;NDUFS5;UQCRC1;ATP6V1H;NDUFV2;ATP6V0C*
**CLL versus Low-count CLL-type MBL**	downregulation	Primary immunodeficiency	8|38	5.03E-04	0.0095115	*DCLRE1C;CD40LG;PTPRC;CD8B;CD3E;IL2RG;IL7R;CD3D*
**CLL versus Low-count CLL-type MBL**	downregulation	RNA transport	18|186	0.005983	0.06653043	*DDX20;PHAX;SNUPN;SRRM1;EEF1A1;PNN;EIF2S3;SUMO1;EIF3I;SUMO2;EIF4EBP2;EIF3H;EIF3E;EIF3F;EIF4E;EIF3D;EIF4G2;EIF3A*
**CLL versus Low-count CLL-type MBL**	downregulation	Spliceosome	15|150	0.008603	0.08541271	*ISY1;SF3A3;BUD31;CDC5L;LSM5;CRNKL1;HNRNPK;SNW1;SYF2;PCBP1;HNRNPC;SNRPC;SLU7;BCAS2;CTNNBL1*
**CLL versus Low-count CLL-type MBL**	downregulation	Hematopoietic cell lineage	11|99	0.010863	0.10413939	*CD2;HLA-DMA;CD8B;HLA-DPB1;CD3G;IL7R;CD3E;HLA-DOB;IL6R;CD3D;HLA-DQB1*

Supplementary Table 9. Types of differential alternative splicing events in pairwise comparisons between Low-count CLL-type MBL, CLL-type MBL and CLL.

	Differential splicing	KEGG Term	Overlap	P-value	Adjusted P-value	Genes
**CLL-type MBL versus Low-count CLL-type MBL**	underrepresentation	oxidative phosphorylation	4|133	0.0394588	0.366992607	*NDUFS5;SDHC;UQCR10;ATP6V1F*
	underrepresentation	NF-kappa B signaling pathway	6|104	0.00050379	0.088164097	*SYK;LCK;TRAF5;LTA;LY96;LAT*
	underrepresentation	Spliceosome	5|150	0.01497173	0.291117035	*PRPF4;HNRNPK;SNRNP27;SNRPB2;U2AF1L4*
**CLL versus CLL-type MBL**	underrepresentation	RNA transport	2|186	0.02962923	0.144169418	*SUMO1;NUP153*
	overrepresentation	B cell receptor signaling pathway	9|81	0.00070761	0.042263446	*PPP3CA;PPP3CB;SYK;MAPK1;LILRB1;NFATC1;LILRB2;LILRB4;VAV2*
	overrepresentation	NF-kappa B signaling pathway	8|104	0.01301895	0.270097452	*SYK;LCK;TRAF5;LTA;LY96;TRAF1;MAP3K7;LAT*
**CLL versus Low-count CLL-type MBL**	underrepresentation	RNA transport	3|186	0.03062266	0.620266523	*SUMO1;THOC7;PAIP1*
	overrepresentation	B cell receptor signaling pathway	6|81	0.0108989	0.2225655	*PPP3CA;PPP3CB;MAPK1;LILRB1;NFATC1;VAV2*
	overrepresentation	MAPK signaling pathway	15|294	0.00350299	0.138951895	*HSPA8;FLT3LG;NFATC1;EFNA4;CDC25B;NFKB2;TGFBR2;ELK4;PPP3CA;PPP3CB;CASP3;MAPK1;MAP2K7;MAP3K7;ATF4*

Supplementary Table 10. Association analysis between different types of biological features and the size of the CLL cell clone in all cases of the present cohort.

Data type	Parameter	Category	Mean (SD)	p value
**Basic demographics**	Gender	Female	1668.4 (2055.24)	0.309
		Male	600.2 (1013.73)	
	Age	(Kendall's tau coefficient)	0.2	0.042
**Immunogenetic data**	Clonality profile	Monoclonal	1150.8 (1433.1)	0.008
		Biclonal	2540.9 (3458.3)	
		Oligoclonal	67.4 (186.3)	
	Light chain isotype	Kappa	956.9 (1558.1)	0.32
		Kappa/Lambda	0.4 (-)	
		Lambda	914.8 (969.4)	
**BcR-related functional data**	BcR IG reactivity (autoantigens)	C1	95.5 (-)	0.207
		C2	351.2 (228.2)	
		C3	1717 (1661.4)	
	BcR IG reactivity (microbes)	No	1178.1 (1561.2)	0.756
		Yes	145.1 (47.3)	
	BcR autonomous signaling	Absent	871 (826.3)	0.55
		Weak	2193.6 (2833.8)	
		Strong	1969 (1707.6)	
**Genetic and transcriptomic data**	Genetic drivers	No	829.9 (1435.7)	0.023
		Yes	1879.4 (1580.9)	
	Transcriptomic profile	C1	3178 (1443)	0.03
		C2	164.1 (319.7)	
		C3	1817.6 (1995.2)	
	BcR signaling pathway	C1	0.1 (NA)	0.002
		C2	2860.7 (1605.4)	
		C3	159.2 (307.7)	
